# Efficacy and Safety of CAR-T Therapy for Relapse or Refractory Multiple Myeloma: A systematic review and meta-analysis

**DOI:** 10.7150/ijms.46811

**Published:** 2021-02-18

**Authors:** Qin Yang, Xin Li, Fangrong Zhang, Qiaohui Yang, Wen Zhou, Jing Liu

**Affiliations:** 1 Department of Hematology, The Third Xiangya Hospital, Central South University, Changsha, Hunan, P.R. China.; 2 Key Laboratory of Carcinogenesis and Cancer Invasion, Ministry of Education; Key Laboratory of Carcinogenesis, National Health and Family Planning Commission, Cancer Research Institute, School of Basic Medical Science, Central South University, Changsha, Hunan, P.R. China.; 3 Cancer Research Institute, Central South University, Changsha, Hunan, P.R. China.; 4 Institute of Reproductive and Stem Cell Engineering, School of Basic Medical Science, Central South University, Changsha, Hunan, P.R. China.

**Keywords:** RRMM, CARs, CAR-T therapy, antigens, BCMA, co-stimulatory domain, systematic review, meta-analysis

## Abstract

**Background:** Multiple myeloma (MM) is incurable in spite of recent treatment improvements, highlighting the development of new therapies. Chimeric antigen receptor (CAR) T-cell therapy has dramatically changed the therapeutic effectiveness in high-risk B-cell malignancies. For relapsed/refractory multiple myeloma (RRMM), preclinical evaluations of CAR-T therapy have shown promising efficacy, thus various active clinical trials are under way. Herein, we conducted this review to summarize efficacy and safety of CAR-T therapy and provide more evidence to guide clinical treatments.

**Method:** We systematically searched literature based on databases (PubMed, EMBASE, Cochrane Central Register of Controlled Trials), and conference abstracts reported from American Society of Hematology (ASH), European Hematology Association (EHA) and American Society of Clinical Oncology (ASCO), in addition to other sources (www.clinicaltrials.gov, article citations). Data assessed efficacy and safety of CAR-T therapy in patients with RRMM were extracted and evaluated, and then systematically analyzed by Comprehensive Meta-analysis 3.0 (CMA 3.0).

**Results:** A total of 23 studies including 350 participants from different countries, diagnosed as RRMM and treated with CAR-T therapy (containing 7 antigens targeted by CARs) were combined. In summary, we discovered the pooled overall response rate (77%), complete response rate (37%) and minimal residual disease (MRD) negativity rate within responders (78%). Furthermore, the pooled relapse rate of responders was 38% and median progression-free survival was 8 months. The pooled survival rate was 87% at last follow-up (median, 12 months). In addition, the pooled grade 3-4 rates of cytokine release syndrome (CRS) and neurologic toxicities (NT) were 14% and 13%, respectively.

**Conclusion:** Our study suggests that CAR-T therapy has demonstrated efficacy and safety in RRMM patients. BCMA-targeted CAR-T and anti-BCMA contained regimen have shown better efficacy.

## Introduction

Multiple myeloma (MM) is a B-cell malignancy characterized by the aberrant expansion of clonal plasma cells and clinically manifested by hypercalcemia, renal dysfunction, anemia, and bone lesions. It accounts for 1-2% of all malignancies and ranks second among the hematological malignancies, just following non-Hodgkin lymphoma 1. With the advent of proteasome inhibitors, immunomodulatory drugs and monoclonal antibodies, the overall survival of myeloma patients has been improved significantly over the last few years. In spite of recent development of therapeutic regimens, most patients will inevitably relapse and need salvage regimens, which are generally associated with a reduced duration of responses, highlighting the development of new therapies.

Chimeric antigen receptors (CARs) are engineered receptors that combine an antigen-recognition domain and T-cell signaling domains 2-4. Expressing a CAR, T cells can specifically recognize a desired antigen, which was first described in the late 1980s 5, 6. It was a highly effective form of adoptive cell therapy, and was approved by FDA in B cell acute lymphoblastic leukemia or large B cell lymphoma patients with the desirable remission rates 7, 8. The impressive results provided rationales for developing CAR-T against MM. Preclinical evaluations of CAR-T cells showed promising efficacy for MM, especially for relapsed/refractory setting (RRMM). The first in-human trial on anti-BCMA CAR-T therapy was conducted by Brudno, which achieved a high response rate 9. Subsequently, various ongoing clinical trials utilizing CAR-T technology have been performed to target myeloma antigens such as B cell maturation antigen (BCMA), CD19, CD138 and immunoglobulin light chains 10-13. Up to now, the comprehensive analysis about this novel therapy on RRMM based on the current data has not been performed yet. And evaluation of the benefits of CAR-T therapy in patients with RRMM is necessary. Herein, we systematically review the current literature and report the results of a meta-analysis. It included a total of 23 studies incorporating 350 participants diagnosed as RRMM and treated with CAR-T therapy from 2016 to 2019, to study the efficacy and safety of CAR-T therapy and provide better understanding of this new treatment strategy.

## Methods

### Study design and search process

This systematic review and meta-analysis followed the Preferred Reporting Items for Systematic Reviews and Meta-Analyses (PRISMA) guidelines in the Cochrane Handbook 14, 15. Research articles that assessed the efficacy and safety of CAR-T therapy in patients with RRMM were the major objectives.

The search process was conducted based on two major resources as follows. Firstly, literature published on PubMed, EMBASE, Cochrane Central Register of Controlled Trials, and other sources (www.clinicaltrials.gov, article citations). Secondly, conference abstracts reported from American Society of Hematology (ASH), European Hematology Association (EHA) and American Society of Clinical Oncology (ASCO).

The following keywords were used to construct the search strategy: (chimeric antigen receptor OR car t therapy OR car t immunotherapy OR car t cell OR modified or engineered t cell) AND (multiple myeloma OR myeloma OR multiple myeloma relapse OR multiple myeloma refractory). Additionally, we boosted our literature search through a manual search of the reference lists of eligible articles.

### Eligibility criteria

Two authors independently screened and judged the eligibility of identified articles, and disagreements were resolved by consensus. The included studies had to conform to the following criteria: (1) enrollment of subjects (age≥18 years) receiving CAR-T therapy, no matter what antigens CAR-T cells targeted; (2) evaluation of the efficacy and safety of CAR-T therapy; (3) one or more outcomes such as response, relapse or survival, and adverse events were reported.

### Data extraction and quality assessment

The following data were extracted by 2 independent authors: the name of the first author, the year of publication, registration number, sample size, age and country of participants, lines of prior treatment, autologous stem cell transplantation (ASCT) before CAR-T, CAR construct, CAR antigens, T cell origin and subset, lymphodepletion, CAR-T doses, outcomes such as response, relapse or survival, and adverse events, duration of follow-up. The modified Institute of Health Economics (IHE) risk of bias tool 16 was used to perform the quality assessment of the included studies.

### Statistical analysis

The following outcomes were to be measured, such as overall response, complete response, MRD negativity within responders, relapse, survival and adverse events. Data of each study were pooled to estimate the efficacy and safety of CAR-T therapy by using a random - effects models with the DerSimonian and Laird random-effects method (Comprehensive Meta-analysis 3.0, Englewood, USA). Heterogeneity was evaluated on Q-statistic and *I^2^* statistics 17 and values of 25, 50 and 75% were used to represent low, medium and high quality respectively 18. The source of heterogeneity would be explored if there was considerable heterogeneity. The evaluation of publication bias was performed by funnel plot and Egger test.

## Results

### Literature search results

**Figure 1:**
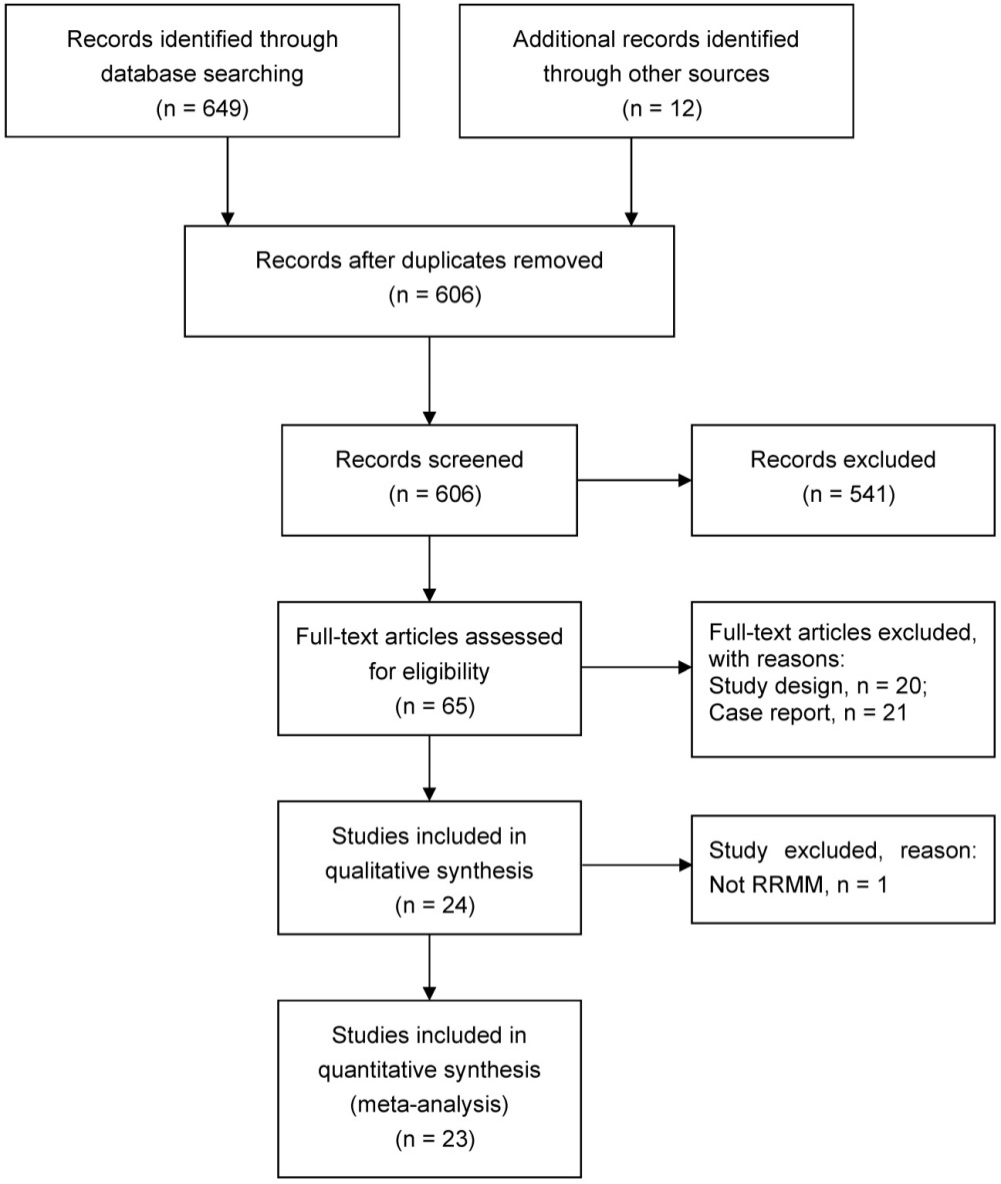
PRISMA flowchart of study search.

The database search identified 661 potentially eligible studies. After screening titles/abstracts and retrieving full-text articles, a total of 23 studies met the inclusion criteria, which included 350 participants from multiple centers diagnosed as RRMM and treated with CAR-T therapy from 2016 to 2019 9, 11-13, 19-37 (Figure 1).

### Quality assessment

The modified IHE tool included assessment of the study objective, design, study population, intervention and co-interventions, outcome measures (e.g., blinding, incomplete outcome data such as participants lost to follow-up, selective outcome reporting), statistical analysis, results, conclusions and conflicts of interest. Each item was scored as high risk, moderate risk or low risk of bias. The evaluation was made independently by two authors based on the criteria provided for the modified IHE risk of bias tool for interventional study designs. Disagreements were resolved by discussion. All the studies were assessed as low and moderate risk. Table S1 stated the details of the quality of the included studies.

### Study characteristics

**Figure 2:**
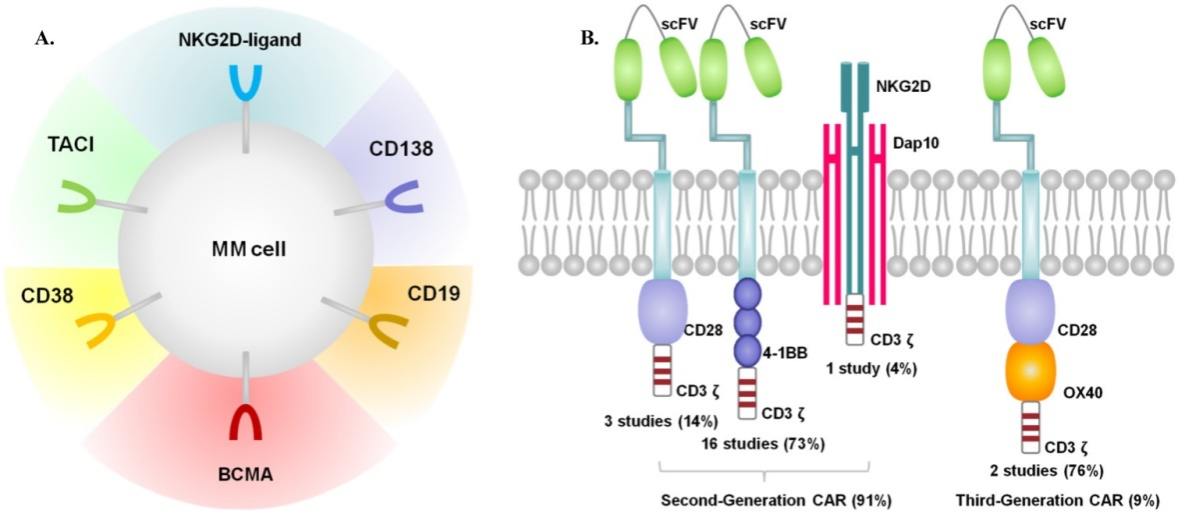
Summary of target antigens (A) and CARs (B) included in our review.

All of the 23 included studies, published between 2016 and 2019, were single-arm early phase studies. Most of the enrolled participants were middle-aged or elderly, with performance status 0-2 assessed by Eastern Cooperative Oncology Group (ECOG). All patients were diagnosed with RRMM and received multiple prior lines of treatments (proteasome inhibitors, immunomodulatory drugs, monoclonal antibodies or ASCT). Among the 23 studies, 2 studies had no statements on prior treatments 13, 26. Most participants underwent ASCT as part of previous treatments. 1 study performed ASCT concurrently with CAR-T therapy 11, and data with ASCT were absent within 7 studies. Most studies performed lymphodepletion by fludarabine and cyclophosphamide. Melphalan was used in 1 study 11, and cyclophosphamide in 2 studies 20, 37.The lymphodepletion was not performed in 1 study 36, and the data were not available in 2 studies 12, 13. T cells were genetically modified to express a CAR by using γ-retroviruses, lentiviruses, or transposon systems. The origin of T cells in most studies were autologous, while 1 study with both autologous and allologous T cells 35, and 5 studies with T cells of unknown origin. The characteristics of the included studies were shown in Table 1. Figure 2 presented the target antigens and CARs included in our review.

### Outcomes of meta-analysis

#### Basic pooled proportions of patients with CAR-T therapy

**Figure 3:**
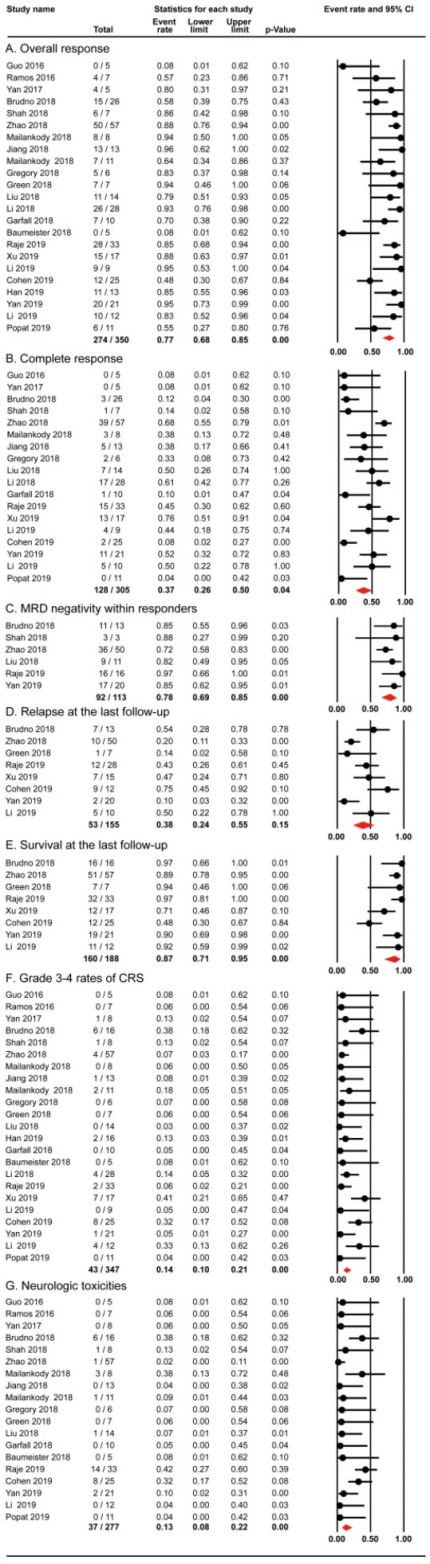
The pooled proportions of efficacy and safety outcomes (A. Overall Response; B. Complete Response; C. MRD negativity within responders; D. Relapse at last follow-up; E. Overall Survival at last follow-up; F. Grade 3-4 rates of CRS; G. NT) for RRMM with CAR-T.

All the 23 studies reported the outcomes of overall response. 274 out of 350 patients in the 23 studies achieved overall response, with the pooled proportion being 77% (95% CI: 68-85; *I^2^*=57.458%; *p*< 0.01; Figure 3A). 128 out of 305 patients in 18 studies achieved complete response. The random-effects pooled proportion was 37% (95% CI: 26-50; *I^2^*=68.271%; *p*< 0.01; Figure 3B). In terms of MRD negativity within responders, 92 out of 113 participants in 6 studies obtained MRD negativity. The pooled proportion was 78% (95% CI: 69-85; *I^2^*=31.394; *p<* 0.01; Figure 3C). Our analysis revealed the overall response rate (77%) and MRD negativity rate within responders (78%) were impressive, although the number of patients with MRD data was not high. Our pooled complete response rate was 37%, and slightly lower than the best reported rate 43% 38.

Furthermore, 8 studies evaluated relapse and survival outcomes at the last follow-up. The pooled proportion of relapse was 38% (95% CI: 24-55; *I^2^*=67.280; *p*< 0.01; Figure 3D) and the median progression-free survival was 8 months. The pooled proportion of survival (median follow-up, 12 months) was 87% (95% CI: 71-95; *I^2^*=73.904; *p*< 0.01; Figure 3E).

The important toxicities of cytokine release syndrome (CRS) and neurologic toxicities (NT) were also evaluated in this meta-analysis. 43 out of 347 patients in the 23 studies experienced grade 3-4 rates of CRS with the pooled proportion being 14% (95% CI: 10-21; *I^2^*=34.223; *p>* 0.01; Figure 3F). The pooled proportion of NT in 19 studies was 13% (95% CI: 8-22; *I^2^*=50.454; *p*< 0.01; Figure 3G). However, the criteria for CRS and NT grading varied previously and there has been a consensus now 39-41. Our analysis showed the pooled rates of serious adverse events (CRS and NT) were relatively infrequent (14% and 13%).

### Multiple factors associated with overall response rate and adverse events rates

#### The subgroup analysis of overall response and adverse events by country

13 studies were conducted in Western countries and 10 studies in Eastern countries. Further subgroup analysis revealed that the 86% (95%CI: 76-92) pooled overall response rate in Eastern countries were statistically different from the 69% (95%CI: 56-79) pooled overall response rate in Western countries. As to adverse events, there was no difference in grade 3-4 rates of CRS between Eastern and Western countries: 14% (95%CI: 8-24) versus 14% (95%CI: 8-23). A higher NT rate was observed in Western countries in comparison to Eastern countries: 23% (95%CI: 15-34) versus 6% (95%CI: 2-13). Based on the analysis above, it was presumable that the antimyeloma activity of CAR-T therapy in Eastern patients may be better than Western patients. The grade 3-4 rates of CRS showed no difference between them, while the NT was more prone to Western, indicating the possible effects of racial difference on the efficacy of CAR-T. However, the results should be interpreted with caution. Many factors including patient selection may contribute to the better response of Eastern patients. Additionally, the lower rate of NT events in Eastern patients may be related to reporting bias. Thus, more studies were needed to further understand the underlying mechanisms of the differences.

### The subgroup analysis of overall response and adverse events by trial site

11 studies of 199 participants were performed in multiple centers, while 12 studies of 151 participants in single centers. We carried out a subgroup analysis by using trial site as a moderator. In conclusion, the pooled overall response rate of multiple centers was 80% (95%CI: 66-88) and showed no significant difference when compared with a 75% (95%CI: 60-86) overall response rate in single centers. There was also non-significant difference with regard to adverse events rates.

### The subgroup analysis of overall response and adverse events by trial status

7 studies were completed and 16 studies are still ongoing. Our subgroup analysis of trial status categorized by completed and ongoing revealed non-significant difference in overall response rate: 72% (95%CI: 51-86) versus 80% (95%CI: 68-88), so did the adverse events rates.

### The subgroup analysis of overall response and adverse events by scFvs origin

The single-chain variable fragments (scFvs) originated from murine (7 studies), llama (3 studies) and human (9 studies) in the included studies. Further subgroup analysis demonstrated that the pooled overall response rate of human scFvs origin was 74% (95%CI: 53-87) and showed no significant difference when compared with the 83% (95%CI: 72-90) overall response rate of non-human scFvs origin. The pooled CRS of grade 3-4 rates and NT rates showed no significant differences between human and non-human scFvs origin.

### The subgroup analysis of overall response and adverse events by co-stimulatory domain

The main co-stimulatory molecules were CD28 and 4-1BB in the included studies. As defined, one co-stimulatory signaling domain is added in second-generation CARs, and two co-stimulatory signaling domains are added in third-generation type. Specially, NKG2D is categorized as second-generation type, which can naturally bind to the natively-encoded adaptor protein DAP10 and then provide a co-stimulatory signal upon ligand binding 19. Based on the above classification, 20 studies used the second-generation CARs structure, and only 2 studies used the third-generation type.

We conducted subgroup analysis of co-stimulatory domains, which were categorized by 4-1BB- and CD28- contained second-generation CAR-T. The pooled overall response rate was 81% (95%CI: 71-89) and 72% (95%CI: 45-90) for 4-1BB- and CD28- contained second-generation CAR-T respectively. However, the difference wasn't significant statistically, as well as the adverse events rates. Therefore, we may get that 4-1BB- and CD28-contained second-generation CAR-T showed the same efficacy and safety.

Then we performed our subgroup analysis of second-generation and third-generation CARs. It demonstrated that the pooled overall response rate of second-generation CARs was 78% (95%CI: 68-86) and showed no significant difference when compared with a 65% (95%CI: 26-91) overall response rate of third-generation type. The adverse events rates were observed no difference as well. Theoretically, third-generation CARs were designed to enhance the efficacy with the advances in the field of CAR-T cell engineering. Nevertheless, some preclinical studies discovered that third-generation CARs had no difference or even worse function in comparison to second-generation CARs 42-45. Our result showed the same activity between third-generation and second-generation CAR-T therapy, which deserved exploration for its reasons.

### The subgroup analysis of overall response and adverse events by antigens targeted by CAR

Multiple antigen targets were included in our analysis. 19 studies used single-target CAR (15 BCMA, 1 CD19, 1 CD138, 1κ light chain, 1 NKG2D-ligands), and 4 studies used dual-target CAR (2 CD19 and BCMA, 1 BCMA and CD38, 1 BCMA and TACI).

We further conducted subgroup analysis by using the antigens as a moderator. The pooled overall response rate of BCMA-targeted CAR-T was 82% (95%CI: 72-89) when compared with non-BCMA-targeted one (43%, 95%CI: 18-72). The BCMA-targeted CAR-T had higher overall response rate than non-BCMA-targeted type (*p<* 0.05). Meanwhile, the adverse events rates revealed no significant difference. To sum up, BCMA-targeted CAR-T showed better efficacy than non-BCMA-targeted type, which may give a reasonable evidence to optimize the CAR-T therapy by choosing BCMA-targeted one.

Furthermore, we compared anti-BCMA contained regimen with anti-BCMA uncontained one. It turned out that the 81% (95%CI: 73-88) pooled overall response rate of anti-BCMA contained regimen were statistically different from the 43% (95%CI: 18-72) pooled overall response rate of anti-BCMA uncontained regimen. Meanwhile, the adverse events rates were observed no difference. By the subgroup analysis, we concluded that anti-BCMA contained regimen had higher overall response rate without bringing additional toxicities. Nevertheless, the number of non-BCMA targets in clinical trials was much smaller than BCMA, and non-BCMA targeted approaches were more of the time period during which they were done (early in CAR-T development). These may under- or over-estimate the pooled proportions.

Then we analyzed the pooled overall response rate of single-target CAR-T (77%, 95%CI: 66-85) when compared with dual-target CAR-T (80%, 95%CI: 55-93). The difference wasn't significant statistically, so did the adverse events rates. Theoretically, combining CAR-T cells with different targets in a cocktail infusion may combat antigen loss and resistance of CAR-T 46, and show better effectiveness than single-target one. But there was no difference between dual-target CAR-T therapy and single-target one, which need further investigation to testify this outcome and explain the reasons. All the subgroup analysis were demonstrated in the supplementary Figure 1-3.

### Publication bias

Risk of publication bias was evaluated by funnel plot and Egger's tests. No evidence of potential publication bias was revealed for overall response, complete response, relapse and overall survival at last follow-up by visual inspection and Egger's tests. However, considerable publication bias was identified by visual inspection and Egger's tests regarding the outcomes of MRD negativity within responders, grade 3-4 rates of CRS and NT (Figure S4).

## Discussion

Nowadays, cancer therapy has already transitioned from conventional chemotherapy to specific immune-based therapeutic strategies. As a state-of-the-art method, CAR-T therapy has achieved remarkable success in refractory hematological malignancies. Clinical trials of CD19-targeted CAR-T therapy were tested in acute and chronic leukemia with a 70%-90% response rate 47. As for CAR-T therapy in MM, some previous studies have demonstrated the therapeutic potential. In this review, we assessed the efficacy and safety of CAR-T therapy based on 23 studies including 350 participants with RRMM globally. The analysis above revealed CAR-T therapy showed promising outcomes with tolerable toxicities in RRMM patients. Furthermore, BCMA-targeted CAR-T and anti-BCMA contained regimen contributed to better efficacy.

The design of co-stimulatory domains in CARs is a critical step to strengthen the function of T cells against antigens 48. The co-stimulatory molecules are generally derived from either the CD28 receptor family (CD28, ICOS) or the tumor necrosis factor receptor family (4-1BB, OX40, CD27) 49. And CD28 and 4-1BB have been most frequently used in clinical trials 3, 50. The CAR-T therapy could produce more robust cytokines and enhance cytolytic capacity by adding CD28, 4-1BB, OX40, and other immune T-cell co-stimulators 3, 50-54. The second-generation CARs have confirmed more supreme cytokine productivity and antitumor activity in mice in comparison to the first-generation ones 3. It remains to be investigated whether other co-stimulatory molecules will exceed the well-established CD28 and 4-1BB domains. In addition, the profound understanding of influences on distinct domains is urgent so as to optimize CAR-T design and therapy.

MM is genetic and phenotypic heterogeneity. And this phenotypic heterogeneity includes differences in cell-surface antigen expressions 55. Therefore, the choice of target antigens is crucial to determine antimyeloma activity and toxicity of CAR-T therapy. As observed in clinical trials involving anti-CD19 CAR-T therapy, hypogammaglobinemia was generated by destructing all B cells expressing CD19 56, 57, regardless of normal or malignant one 48. Thus, the targeted antigen should be absent on important normal tissues. Unfortunately, no plasma cells antigens were found to be strongly and uniformly expressed on all malignant plasma cells and not on normal cells. Further investigations should focus on this part to enhance the specificity of CAR-T therapy without increasing toxicities. In our review, we included multiple antigens targeted by CAR, most of which were BCMA. BCMA is known as CD269 and TNF receptor superfamily 17 (TNFRSF17) 58, and expressed in plasma cells and myeloma cells rather than in normal tissues and hematopoietic stem cells 46. BCMA was recently reported to be uniformly expressed in most cases of MM by immunohistochemistry and flow cytometry 59, indicating that BCMA targeted CAR-T products may exert antimyeloma activity without serious adverse effects theoretically. In fact, plenty of BCMA targeted CAR-T products have been applied in active clinical trials. Several MM patients with infusion of anti-BCMA CAR-T have obtained objective responses, which is expected to be approved for clinical therapy in managing RRMM soon after. Other antigen targets, CD19, CD138, κ light chain and NKG2D-ligands, also have shown activities in RRMM. Further researches should aim to identify new targets for CAR-T therapy and optimize this strategy regarding tandem CAR-T or dual receptors within one T cell.

Toxicities such as CRS and NT restricted the widespread use of CAR-T therapy. CRS, the most important CAR-T toxicity, is an inflammatory syndrome caused by multiple cytokines produced by the CAR T cells themselves and other cells. It features hypotension, fevers, and tachycardia among many other abnormalities 48, 60. NT are complex syndromes including encephalopathy, cognitive defects, dysphasias, seizures and cerebral edema, the pathogenesis of which remains poorly understood 60. Multiple factors can result in CAR-T toxicity, including early and peak levels of certain cytokines, peak blood CAR-T cell levels, CAR-T cell doses, CAR design, patients' disease burden and so on. 60. Toxicity management typically includes supportive care and immunosuppression with tocilizumab or corticosteroids 40, 61, 62. In the future, further mechanistic understanding of these toxicities is an important area to improve the efficacy-to-toxicity ratio of CAR-T therapy.

In the section above, we have emphasized the therapeutic potential of CAR-T for MM by the promising early results, but this approach still has some limitations. Factors precluding durable remissions following CAR-T therapy include CAR-T cell manufacturing issues, limited CAR-T cell expansion and/or persistence, various resistance mechanisms and toxicities 63. Firstly, this novel promising therapy requires a CAR T cell product to be successfully manufactured infused, activated and expanded, and then effectively mediates a cytotoxic response. Issues outlined above present barriers to effective efficacy and need to be improved. Secondly, poor persistent efficacy and resistance to CAR-T were observed in our analysis, which may be explained by immune escape due to target antigen loss or modulation 63. Thirdly, selectivity of patients and cost-effectiveness should not be overlooked to expand patient access to CAR-T therapy. As for anti-BCMA CAR-T therapy, the possible reasons may include soluble BCMA blocking it from getting to the target and downregulation of the antigen's expression on malignant plasma cells over time. Based on these limitations, this novel treatment should be rationally applied in clinical practice.

In summary, our meta-analysis demonstrated impressive results of CAR-T cells, especially the anti-BCMA type, which can induce remarkable responses in highly pretreated RRMM patients. CAR-T therapy for MM is still at an early stage and more profound mechanisms are needed to be elucidated. In the future, phase 3 clinical trials should focus on investigating the efficacy and safety of CAR-T therapy so as to provide more evidence for more focused analysis of specific subgroups and make individualized treatments in subsequent studies. In addition, further researches should also optimize the design of CARs, explore different CAR-T products, and combine them with immunomodulatory drugs, checkpoint inhibitors, other CAR T cells, as well as gene-edited cellular products to enhance the safety and efficacy of this approach.

### Strength and limitations

This systematic review and meta-analysis has the following strengths: we used a predefined search strategy and conducted data extraction and quality evaluation by two independent reviewers to minimize reviewer bias. Moreover, we systematically analyzed publication data about scFvs origin, co-stimulatory domains and antigens targeted by CAR, which was firstly evaluated in RRMM with CAR-T therapy. We conducted subgroup analysis by using country of enrolled participants, trial site and trial status as a moderator. These conclusions may contribute to making evidence-based decision and encouraging further research.

Nevertheless, our systematic review and meta-analysis has the following limitations. Firstly, quality of the included studies was assessed as considerable risk and statistical heterogeneity. Without the uniform criteria and baseline characteristics, data were heterogeneous. Moreover, several studies are still ongoing, and only publish preliminary outcomes, predicating the discrepancy of median follow-up. In a conclusion, a considerable risk of bias exists. Secondly, with a small number of articles, the estimate of subgroup analysis may under- or over-estimate the pooled proportions. Furthermore, we did not analyze the data on specific subgroups, including disease status, high-risk features, prior ASCT treatment, CAR-T doses, BCMA expression and CAR-T persistence due to lack of sufficient information. As the length of follow-up differs, the duration of response didn't be analyzed. Thirdly, no large-scale randomized controlled trials of CAR-T in RRMM have been reported. How much profit do this new strategy bring remains to be investigated.

## Conclusion

Our study suggests that CAR-T therapy has demonstrated efficacy and safety in RRMM patients. BCMA-targeted CAR-T and anti-BCMA contained regimen have shown better efficacy. Our findings call for conducting large-scale randomized trials to obtain more evidence of CAR-T therapies in MM.

## Supplementary Material

Supplementary figures and tables.

## Figures and Tables

**Table 1:** Characteristics of included studies

Reference	Country	Sample size	Median age (range)	Sex (Male/Female)	Lines of Prior treatment	ASCT before CAR-T	LD	Antigen	CAR construct: vector/costimulatory molecule/scFv species	T cell origin	T cell subset	BCMA positivity requirement at enrollment	CAR-T dose	Follow-up
Brudno 2018	Western	26	(18-70)	13/13	10 (3-19)	85%	Flu/Cy: 30mg/m^2^ /300mg/m^2^ daily on day -5 to -3	BCMA	γ-retrovirus/CD28/murine	Autologous	CD4/CD8	>50%	0.3-9.0x10^6^/Kg	Median 20 weeks
Shah 2018	Western	8	64 (54-70)	NA	9 (4-17)	88%	Flu/Cy: 30mg/m^2^ /300mg/m^2^ daily for 3 days	BCMA	Lentivirus/4-1BB/murine	Autologous	NA	Dose-escalation: >50%	150x10^6^	Median 16 (2-27) weeks
Zhao 2018	Eastern	57	54 (27-72)	34/23	3 (1-9)	18%	Cy:300 mg/m^2^ on day -5 to -3	BCMA	Lentivirus/4-1BB/Llama	Autologous	Unselected	Required (cutoff NR)	Median 0.5 (0.07-2.1)x10^6^/Kg	Median 32 (2.8-82.8) weeks
Mailankody 2018	Western	44	53 (36-66)	NA	10 (4-15)	88%	Flu/Cy: 30mg/m^2^ /300mg/m^2^ on days -7 to -2	BCMA	Lentivirus/4-1BB/human	NA	NA	NR	50-150x10^6^	Median 5 (4-13) weeks
Jiang 2018	Western	16	55 (39-67)	NA	4 (2-10)	56%	Flu/Cy: 20-25mg/m^2^ /300- 500mg/m^2^ daily for 2-4 days	BCMA	NR/4-1BB/human	Autologous	NA	≥50%	0.5-1.8x10^8^	Median 8 (4-36) weeks
Mailankody 2018	Western	11	NA	NA	6 (4-14)	100%	Flu/Cy: 30 mg/m^2^ /300 mg/m^2^ for 3 days	BCMA	Retrovirus/4-1BB/human	Autologous	NA	Required (cutoff NR)	72-818x10^6^	>40 weeks
Gregory 2018	Western	12	NA	NA	3-9	NA	Flu/Cy: 30 mg/m^2^/300mg/m^2^ for 3 days	BCMA	Transposon/4-1BB/human	Autologous	NA	Not required	48-430x10^6^	>12 weeks
Green 2018	Western	7	63 (49-76)	NA	8 (6-11)	71%	Flu/Cy	BCMA	Lentivirus/4-1BB/human	Autologous	CD4/CD8	≥5%	5-15x10^7^	Median 16 (2-26) weeks
Liu 2018	Eastern	17	NA	NA	>2	NA	Flu/Cy: 25 mg/m^2^ /300mg/m^2^ on days -5 to -3	BCMA	γ-retrovirus/4-1BB/mouse	Autologous	NA	>5%	9x10^6^	>60 weeks
Li 2018	Eastern	28	NA	NA	NA	NA	Flu/Cy	BCMA	Lentivirus/CD28/murine	Autologous	NA	Required (cutoff NR)	5.4-25.0x10^6^	Median 40 weeks
Raje 2019	Western	33	60 (37-75)	21/12	7 (3-23)	97%	Flu/Cy: 30mg/m^2^ /300mg/m^2^ daily on day -5 to -3	BCMA	Lentivirus/4-1BB/murine	Autologous	CD4/ CD8	Dose-escalation: ≥50%; dose-expansion: NR	50-800x10^6^	Median 45 (24.8-91.2) weeks
Xu 2019	Eastern	17	55 (35-73)	11/6	5 (3-11)	47%	Flu/Cy: 25 mg/m^2^ daily for 3 days/250/300mg/m^2^	BCMA	Lentivirus/4-1BB/Llama	NA	CD4/ CD8	Required (cutoff NR)	0.21-1.52x10^6^/Kg	Median 60 (1.7-76.4) weeks
Li 2019	Eastern	9	NA	NA	4 (3-5)	NA	Flu/Cy	BCMA	Lentivirus/4-1BB/human	NA	NA	NR	1.0-6.0x10^6^/Kg	Median 9 weeks
Cohen 2019	Western	25	58 (44-75)	17/8	7 (3-13)	92%	Cy: 1.5g/m^2^ or no LD	BCMA	Lentivirus/4-1BB/human	Autologous	CD4/ CD8	Not required	1-50x10^7^	Median 54 weeks
Han 2019	Eastern	16	NA	NA	Median 10	NA	Flu/Cy: 30 mg/m^2^ on days -5 to -3/300-600 mg/m^2^ on days -5 to -4	BCMA	Lentivirus/4-1BB/Llama	Autologous	NA	NR	2-10x10^6^	Median 10 weeks
Garfall 2018	Western	10	61 (48-68)	4/6	6 (2-10)	100%	Melphalan: 140-200 mg/m^2^	CD19	Lentiviral/4-1BB	Autologous	Unselected	NR	1-5x10^7^	>14 weeks
Guo 2016	Eastern	5	58 (48-68)	1/4	11 (5-18)	20%	NA	CD138	Lentivirus/4-1BB/human	Autologous	CD8	NR	Median 7.56 (4.4-15.1)x10^6^	Median 12 weeks
Ramos 2016	Western	8	56.5 (43-69)	3/5	NA	NA	NA	κ light chain	Retroviral/CD28/murine	Autologous	CD4/ CD8	NR	0.2-2x10^8^/m^2^	(6-96) weeks
Baumeister 2018	Western	5	70 (44-79)	3/2	≥5	100%	No LD	NKG2D-ligands	γ-retroviral/ NKG2D-CAR	Autologous	CD4/ CD8	NR	1x10^6^-3×10^7^	(14-107) weeks
Yan 2017	Eastern	8	57 (43-69)	6/2	4 (2-7)	NA	Flu/Cy: 30 mg/m^2^/300 mg/m^2^ on days -5 to -3	CD19 and BCMA	Lentivirus/CD28 + OX40 /murine	Auto/allo-logous	NA	Required (cutoff NR)	25-82×10^6^/kg	Median 5 (2-20 ) weeks
Yan 2019	Eastern	21	58 (49.5-61)	NA	6 (5-8)	14%	Flu/Cy: 30mg/m^2^for 3 days /750mg/m^2^ daily for 1 day	CD19 and BCMA	Lentiviral/4-1BB/human (anti-CD19) murine (anti-BCMA)	Autologous	Unselected	NR	CD19(1×10^6^/Kg) BCMA (1×10^6^/Kg)	Median25.6 (10.3-42.1) weeks
Li 2019	Eastern	12	NA	NA	≥2	17%	Flu/Cy: 25 mg/m^2^/250 mg/m^2^ on days -5 to -3	CD38 and BCMA	NR	NA	NA	BCMA+ or CD38+ ≥ 50%	Median 2.17 (0.5-4.0)×10^6^	Median 22 (6-33) weeks
Popat 2019	Western	11	61 (45-69)	NA	5 (3-6)	73%	Flu/Cy: 30 mg/m^2^ daily/300mg/m^2^ daily for 3 days	TACI and BCMA	Retroviral/CD28 + OX40	NA	NA	NR	15-900×10^6^	NA

**Abbreviations:** ASCT, Autologous stem cell transplantation; CAR-T, Chimeric antigen receptor-T; LD, Lymphodepletion; scFv, single-chain fragment variable; Flu, Fludarabine; Cy, Cyclophosphamide; BCMA, B cell maturation antigen; NA, not available; NR, not reported.

**Table 2:** The pooled proportions of outcomes for RRMM with CAR-T

Outcomes	No. of studies	Patients (n/N)	Pooled proportion (%)	95% CI	Heterogeneity within study (*I^2^*, Q and *p*-value)		
					Q-value	*I^2^* (%)	*p*-value
Overall response	23	274/350	77	68-85	51.714	57.458	<0.01
Complete response	18	128/305	37	26-50	53.579	68.271	<0.01
MRD negativity within responders	6	92/113	78	69-85	7.288	31.394	<0.01
Relapse*	8	53/155	38	24-55	21.394	67.280	<0.01
Overall survival*	8	160/188	87	71-95	26.824	73.904	<0.01
Grade 3-4 rates of CRS	23	43/347	14	10-21	33.446	34.223	>0.01
Neurotoxicity	19	37/277	13	8-22	36.330	50.454	<0.01

*The time means the last follow-up; MRD: minimal residual disease; CRS: cytokine release syndrome.

**Table 3:** Subgroup analysis of overall response and adverse events for RRMM with CAR-T

Subgroups	Overall Response				Grade 3-4 rates of CRS				NT			
	Patients (n/N)	Pooled proportion	95% CI	*p*-interaction	Patients (n/N)	Pooled proportion	95% CI	*p*-interaction	Patients (n/N)	Pooled proportion	95% CI	*p*-interaction
**Country**				<0.05				>0.05				<0.05
Eastern	156/181	86	76-92		23/187	14	8-24		4/117	6	2-13	
Western	118/169	69	56-79		20/160	14	8-23		33/160	23	15-34	
**Trial site**				>0.05				>0.05				>0.05
Single center	113/151	75	60-86		22/157	13	7-23		11/104	10	4-23	
Multiple center	161/199	80	66-88		21/190	15	9-25		26/173	15	7-29	
**Trial status**				>0.05				>0.05				>0.05
Completed	107/141	72	51-86		18/131	17	9-30		9/114	10	4-25	
Ongoing	167/209	80	68-88		25/216	14	8-21		28/163	15	8-27	
**scFvs origin**				>0.05				>0.05				>0.05
Human	61/84	74	53-87		11/84	14	6-27		12/75	16	6-35	
Non-human	190/228	83	72-90		28/225	14	8-23		25/169	14	6-28	
**Co-stimulatory domain**			>0.05				>0.05				>0.05	
4-1BB	209/256	81	71-89		28/260	12	7-19		31/218	13	7-24	
CD28	45/61	72	45-90		10/51	21	8-44		6/23	25	5-66	
**CARs**				>0.05				>0.05				>0.05
2^nd^ generation	254/322	78	68-86		38/316	14	9-20		37/246	15	9-25	
3^rd^ generation	10/16	65	26-91		1/19	8	1-37		0/19	5	1-33	
**Antigen target**				<0.05				>0.05				>0.05
BCMA	223/274	82	72-89		37/268	16	10-23		35/198	19	11-31	
Non-BCMA	11/27	43	18-72		0/27	7	2-25		0/27	7	1-26	
**CAR-T regimen**				<0.05				>0.05				>0.05
Anti-BCMA contained	266/323	81	73-88		43/320	15	10-22		37/250	15	9-25	
Anti-BCMA uncontained	11/27	43	18-72		0/27	7	2-25		0/27	7	1-27	
**CAR-T therapy mode**				>0.05				>0.05				>0.05
Single-target	234/301	77	66-85		37/295	14	9-21		35/225	16	10-27	
Dual-target	40/49	80	55-93		6/52	14	5-34		2/52	6	2-21	

## Abbreviations

CAR: Chimeric antigen receptor (CAR)
RRMM: Relapsed/refractory multiple myeloma
PRISMA: Preferred Reporting Items for Systematic Reviews and Meta-Analyses
MRD: Minimal residual disease
CRS: Cytokine release syndrome
NT: Neurologic toxicities
BCMA: B cell maturation antigen
ASCT: Autologous stem cell transplantation
scFvs: single-chain variable fragments
SLAMF7: CS1 glycoprotein antigen
NKG2D: Natural killer group 2, member D
