# Supplementary material for: Efficacy and Safety of CAR-T Therapy for Relapse or Refractory Multiple Myeloma: A systematic review and meta-analysis
Source: Int J Med Sci. 2021 Feb 18;18(8):1786–97. doi: 10.7150/ijms.46811 (PMC7976586; doi:10.7150/ijms.46811)
Supplement: Supplementary file 1 — Supplementary figures and tables. [file ijmsv18p1786s1.pdf]

Table S1:
Quality assessment by Modified Institute of Health Economics Tool.

Question Text	Brudno 2018	Shah 2018	Zhao 2018	Mailankody 2018	Jiang 2018	Mailankody2018	Gregory 2018	Green 2018	Liu 2018	Li 2018	Raje 2019
Was the aim clearly stated	Yes	Yes	Yes	Yes	Yes	Yes	Yes	Yes	Yes	Yes	Yes
Prospective	Yes	Yes	Yes	Yes	Yes	Yes	Yes	Yes	Yes	Yes	Yes
Multicenter	No	Yes	Yes	Yes	Yes	No	Yes	No	No	unclear	Yes
Consecutive recruitment	Yes	Yes	Yes	Yes	Yes	Yes	Yes	Yes	Yes	Yes	Yes
Eligibility criteria clearly stated	Yes	Yes	Yes	Yes	Yes	Yes	Yes	Yes	Yes	Yes	Yes
Characteristics of patients described	Yes	Partial	Yes	Partial	Partial	Partial	Partial	Partial	Partial	Partial	Yes
Did patients enter the study at a similar point in the disease	Yes	Yes	Yes	Yes	Yes	Yes	Yes	Yes	Yes	Yes	Yes
Intervention of interest described	Yes	Partial	Yes	Partial	Yes	Yes	Yes	Yes	Partial	Partial	Yes
Additional interventions clearly described	No	No	No	No	No	No	No	No	No	No	No
Outcome measures established a priori	Yes	Yes	Yes	Yes	Yes	Yes	Yes	Yes	Yes	Yes	Yes
Outcome assessed blinded to intervention staus	Yes	Yes	Yes	Yes	Yes	Yes	Yes	Yes	Yes	Yes	Yes
All relevant outcomes measured appropriately	Yes	Yes	Yes	Yes	Yes	Partial	Yes	Partial	Yes	Yes	Yes
Relevant outcomes measured before and after the intervention	Yes	Yes	Yes	Yes	Yes	Yes	Yes	Yes	Yes	Yes	Yes

Without performing selective outcome reporting	Yes	Partial	Yes	Partial	Partial	Partial	Partial	Partial	Partial	Partial	Yes
Details of statistical tests reported	Yes	Yes	No	No	No	No	No	No	No	No	Yes
Length of follow-up reported	Yes	Yes	Yes	No	Yes	Yes		Yes	Yes	Yes	Yes
Eestimates of random variability in data analysis of relevant outcomes	No	No	Yes	No	No	No	No	No	No	No	Yes
Adverse events reported	Yes	Partial	Yes	Partial	Yes	Partial	Partial	Partial	Yes	Partial	Yes
Competing interests and sources of support reported	Partial	No	Yes	No	No	No	No	No	No	Partial	Unclear
Risk of bias	Moderate risk	Moderate risk	Low risk	Moderate risk	Moderate risk	Moderate risk	Moderate risk	Moderate risk	Moderate risk	Moderate risk	Low risk

**Question Text**	**Xu 2019**	**Li 2019**	**Cohen 2019**	**Han 2019**	**Guo 2016**	**Ramos 2016**	**Garfall 2018**	**Yan 2017**	**Baumeister 2018**	**Li 2019**	**Popat 2019**	**Yan 2019**
Was the aim clearly stated	Yes	Yes	Yes	Yes	Yes	Yes	Yes	Yes	Yes	Yes	Yes	Yes
Prospective	Yes	Yes	Yes	Yes	Yes	Yes	Yes	Yes	Yes	Yes	Yes	Yes
Multicenter	Yes	No	No	No	No	Yes	No	No	No	Unclear	Yes	No
Consecutive recruitment	Yes	Yes	Yes	Yes	Yes	Yes	Yes	Yes	Yes	Yes	Yes	Yes
Eligibility criteria clearly stated	Yes	Yes	Yes	Yes	Yes	Yes	Yes	Yes	Yes	Yes	Yes	Yes
Characteristics of patients described	Yes	Partial	Yes	Partial	Partial	Yes	Yes	Partial	Partial	Partial	Partial	Yes

Did patients enter the study at a similar point in the disease	Yes	Yes	Yes	Yes	Yes	Yes	Yes	Yes	Yes	Yes	Yes	Yes
Intervention of interest described	Partial	Partial	Yes	Partial	Yes	Yes	Yes	Partial	Partial	Partial	Partial	Yes
Additional interventions clearly described	No	No	No	No	No	No	Yes	No	Yes	No	No	No
Outcome measures established a priori	Yes	Yes	Yes	Yes	Yes	Yes	Yes	Yes	Yes	Yes	Yes	Yes
Outcome assessed blinded to intervention staus	Yes	Yes	Yes	Yes	Yes	Yes	Yes	Yes	Yes	Yes	Yes	Yes
All relevant outcomes measured appropriately	Yes	Yes	Yes	Partial	Yes	Partial	Yes	Yes	Yes	Yes	Yes	Yes
Relevant outcomes measured before and after the intervention	Yes	Yes	Yes	Yes	Yes	Yes	Yes	Yes	Yes	Yes	Yes	Yes
Without performing selective outcome reporting	Partial	Partial	Partial	Partial	Partial	Partial	Partial	Partial	Partial	Partial	Partial	Yes
Details of statistical tests reported	Yes	No	Yes	No	Yes	Yes	Yes	No	Yes	No	No	Yes
Length of follow-up reported	Yes	Yes	Yes	Yes	Yes	Yes	Yes	Yes	Yes	Yes	Yes	Yes
Eestimates of random variability in data analysisof relevant outcomes	Yes	No	Yes	No	No	No	No	No	No	No	No	No
Adverse events reported	Partial	Partial	Yes	Partial	Partial	Partial	Yes	Partial	Yes	Partial	Partial	Yes
Competing interests and sources of support reported	Unclear	Unclear	Yes	No	Yes	Yes	Yes	No	Yes	No	No	Yes

Risk of bias	Low risk	Moderate risk	Low risk	Moderate risk	Moderate risk	Low risk	Low risk	Moderate risk	Low risk	Moderate risk	Moderate risk	Moderate risk

Figure S1 Subgroup analysis of Overall Response for RRMM with CAR-T.

A.

Group by Country	Study name	Total	Event rate	Lower limit	Upper limit	p-Value
Eastern	Guo 2016	0 / 5	0.08	0.01	0.62	0.10
Eastern	Yan 2017	4 / 5	0.80	0.31	0.97	0.21
Eastern	Zhao 2018	50 / 57	0.88	0.76	0.94	0.00
Eastern	Liu 2018	11 / 14	0.79	0.51	0.93	0.05
Eastern	Li 2018	26 / 28	0.93	0.76	0.98	0.00
Eastern	Xu 2019	15 / 17	0.88	0.63	0.97	0.01
Eastern	Li 2019	9 / 9	0.95	0.53	1.00	0.04
Eastern	Han 2019	11 / 13	0.85	0.55	0.96	0.03
Eastern	Yan 2019	20 / 21	0.95	0.73	0.99	0.00
Eastern	Li 2019	10 / 12	0.83	0.52	0.96	0.04
**Eastern**		**156 / 181**	**0.86**	**0.76**	**0.92**	**0.00**
Western	Ramos 2016	4 / 7	0.57	0.23	0.86	0.71
Western	Brudno 2018	15 / 26	0.58	0.39	0.75	0.43
Western	Shah 2018	6 / 7	0.86	0.42	0.98	0.10
Western	Mailankody 2018	8 / 8	0.94	0.50	1.00	0.05
Western	Jiang 2018	13 / 13	0.96	0.62	1.00	0.02
Western	Mailankody 2018	7 / 11	0.64	0.34	0.86	0.37
Western	Gregory 2018	5 / 6	0.83	0.37	0.98	0.14
Western	Green 2018	7 / 7	0.94	0.46	1.00	0.06
Western	Garfall 2018	7 / 10	0.70	0.38	0.90	0.22
Western	Baumeister 2018	0 / 5	0.08	0.01	0.62	0.10
Western	Raje 2019	28 / 33	0.85	0.68	0.94	0.00
Western	Cohen 2019	12 / 25	0.48	0.30	0.67	0.84
Western	Popat 2019	6 / 11	0.55	0.27	0.80	0.76
**Western**		**118 / 169**	**0.69**	**0.56**	**0.79**	**0.00**
**Overall**		**274 / 350**	**0.78**	**0.57**	**0.90**	**0.01**

Event rate and 95% CI

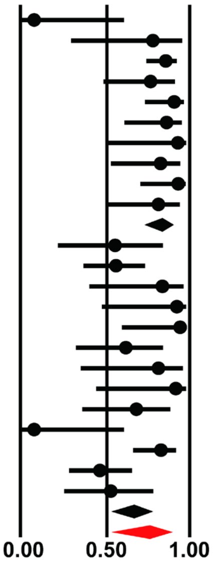

B.

Trial site	Study name	Total	Event rate	Lower limit	Upper limit	p-Value
Multicenter	Ramos 2016	4 / 7	0.57	0.23	0.86	0.71
Multicenter	Brudno 2018	15 / 26	0.58	0.39	0.75	0.43
Multicenter	Shah 2018	6 / 7	0.86	0.42	0.98	0.10
Multicenter	Zhao 2018	50 / 57	0.88	0.76	0.94	0.00
Multicenter	Mailankody 2018	8 / 8	0.94	0.50	1.00	0.05
Multicenter	Jiang 2018	13 / 13	0.96	0.62	1.00	0.02
Multicenter	Gregory 2018	5 / 6	0.83	0.37	0.98	0.14
Multicenter	Liu 2018	11 / 14	0.79	0.51	0.93	0.05
Multicenter	Raje 2019	28 / 33	0.85	0.68	0.94	0.00
Multicenter	Xu 2019	15 / 17	0.88	0.63	0.97	0.01
Multicenter	Popat 2019	6 / 11	0.55	0.27	0.80	0.76
**Multicenter**		**161 / 199**	**0.80**	**0.66**	**0.88**	**0.00**
Single center	Guo 2016	0 / 5	0.08	0.01	0.62	0.10
Single center	Yan 2017	4 / 5	0.80	0.31	0.97	0.21
Single center	Mailankody 2018	7 / 11	0.64	0.34	0.86	0.37
Single center	Green 2018	7 / 7	0.94	0.46	1.00	0.06
Single center	Li 2018	26 / 28	0.93	0.76	0.98	0.00
Single center	Garfall 2018	7 / 10	0.70	0.38	0.90	0.22
Single center	Baumeister 2018	0 / 5	0.08	0.01	0.62	0.10
Single center	Li 2019	9 / 9	0.95	0.53	1.00	0.04
Single center	Cohen 2019	12 / 25	0.48	0.30	0.67	0.84
Single center	Han 2019	11 / 13	0.85	0.55	0.96	0.03
Single center	Yan 2019	20 / 21	0.95	0.73	0.99	0.00
Single center	Li 2019	10 / 12	0.83	0.52	0.96	0.04
**Single center**		**113 / 151**	**0.75**	**0.60**	**0.86**	**0.00**
**Overall**		**274 / 350**	**0.77**	**0.68**	**0.85**	**0.00**

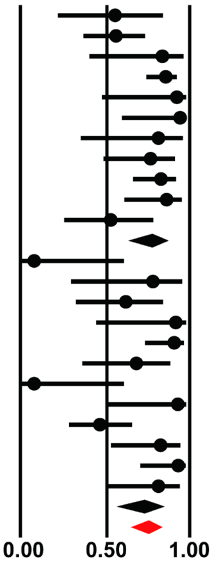

C.

Trial status	Study name	Total	Event rate	Lower limit	Upper limit	p-Value
Completed	Guo 2016	0 / 5	0.08	0.01	0.62	0.10
Completed	Brudno 2018	15 / 26	0.58	0.39	0.75	0.43
Completed	Zhao 2018	50 / 57	0.88	0.76	0.94	0.00
Completed	Garfall 2018	7 / 10	0.70	0.38	0.90	0.22
Completed	Baumeister 2018	0 / 5	0.08	0.01	0.62	0.10
Completed	Xu 2019	15 / 17	0.88	0.63	0.97	0.01
Completed	Yan 2019	20 / 21	0.95	0.73	0.99	0.00
**Completed**		**107 / 141**	**0.72**	**0.51**	**0.86**	**0.04**
Ongoing	Ramos 2016	4 / 7	0.57	0.23	0.86	0.71
Ongoing	Yan 2017	4 / 5	0.80	0.31	0.97	0.21
Ongoing	Shah 2018	6 / 7	0.86	0.42	0.98	0.10
Ongoing	Mailankody 2018	8 / 8	0.94	0.50	1.00	0.05
Ongoing	Jiang 2018	13 / 13	0.96	0.62	1.00	0.02
Ongoing	Mailankody 2018	7 / 11	0.64	0.34	0.86	0.37
Ongoing	Gregory 2018	5 / 6	0.83	0.37	0.98	0.14
Ongoing	Green 2018	7 / 7	0.94	0.46	1.00	0.06
Ongoing	Liu 2018	11 / 14	0.79	0.51	0.93	0.05
Ongoing	Li 2018	26 / 28	0.93	0.76	0.98	0.00
Ongoing	Raje 2019	28 / 33	0.85	0.68	0.94	0.00
Ongoing	Li 2019	9 / 9	0.95	0.53	1.00	0.04
Ongoing	Cohen 2019	12 / 25	0.48	0.30	0.67	0.84
Ongoing	Han 2019	11 / 13	0.85	0.55	0.96	0.03
Ongoing	Li 2019	10 / 12	0.83	0.52	0.96	0.04
Ongoing	Popat 2019	6 / 11	0.55	0.27	0.80	0.76
**Ongoing**		**167 / 209**	**0.80**	**0.68**	**0.88**	**0.00**
**Overall**		**274 / 350**	**0.78**	**0.68**	**0.85**	**0.00**

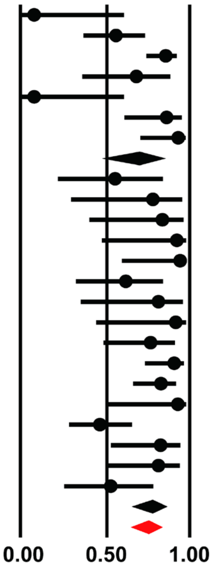

D. scFv origin

Human	Guo 2016	0 / 5	0.08	0.01	0.62	0.10
Human	Mailankody 2018	8 / 8	0.94	0.50	1.00	0.05
Human	Jiang 2018	13 / 13	0.96	0.62	1.00	0.02
Human	Mailankody 2018	7 / 11	0.64	0.34	0.86	0.37
Human	Gregory 2018	5 / 6	0.83	0.37	0.98	0.14
Human	Green 2018	7 / 7	0.94	0.46	1.00	0.06
Human	Li 2019	9 / 9	0.95	0.53	1.00	0.04
Human	Cohen 2019	12 / 25	0.48	0.30	0.67	0.84
**Human**		**61 / 84**	**0.74**	**0.53**	**0.87**	**0.03**
Non-human	Ramos 2016	4 / 7	0.57	0.23	0.86	0.71
Non-human	Yan 2017	4 / 5	0.80	0.31	0.97	0.21
Non-human	Brudno 2018	15 / 26	0.58	0.39	0.75	0.43
Non-human	Shah 2018	6 / 7	0.86	0.42	0.98	0.10
Non-human	Zhao 2018	50 / 57	0.88	0.76	0.94	0.00
Non-human	Liu 2018	11 / 14	0.79	0.51	0.93	0.05
Non-human	Li 2018	26 / 28	0.93	0.76	0.98	0.00
Non-human	Raje 2019	28 / 33	0.85	0.68	0.94	0.00
Non-human	Xu 2019	15 / 17	0.88	0.63	0.97	0.01
Non-human	Han 2019	11 / 13	0.85	0.55	0.96	0.03
Non-human	Yan 2019	20 / 21	0.95	0.73	0.99	0.00
**Non-human**		**190 / 228**	**0.83**	**0.72**	**0.90**	**0.00**
**Overall**		**251 / 312**	**0.80**	**0.71**	**0.87**	**0.00**

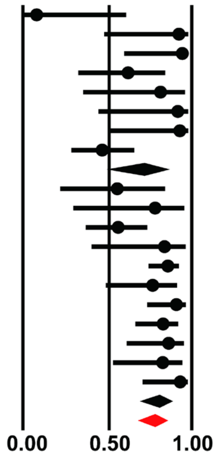

E. Co-stimulatory domain

4-1BB	Guo 2016	0 / 5	0.08	0.01	0.62	0.10
4-1BB	Shah 2018	6 / 7	0.86	0.42	0.98	0.10
4-1BB	Zhao 2018	50 / 57	0.88	0.76	0.94	0.00
4-1BB	Mailankody 2018	8 / 8	0.94	0.50	1.00	0.05
4-1BB	Jiang 2018	13 / 13	0.96	0.62	1.00	0.02
4-1BB	Mailankody 2018	7 / 11	0.64	0.34	0.86	0.37
4-1BB	Gregory 2018	5 / 6	0.83	0.37	0.98	0.14
4-1BB	Green 2018	7 / 7	0.94	0.46	1.00	0.06
4-1BB	Liu 2018	11 / 14	0.79	0.51	0.93	0.05
4-1BB	Garfall 2018	7 / 10	0.70	0.38	0.90	0.22
4-1BB	Raje 2019	28 / 33	0.85	0.68	0.94	0.00
4-1BB	Xu 2019	15 / 17	0.88	0.63	0.97	0.01
4-1BB	Li 2019	9 / 9	0.95	0.53	1.00	0.04
4-1BB	Cohen 2019	12 / 25	0.48	0.30	0.67	0.84
4-1BB	Han 2019	11 / 13	0.85	0.55	0.96	0.03
4-1BB	Yan 2019	20 / 21	0.95	0.73	0.99	0.00
**4-1BB**		**209 / 256**	**0.81**	**0.71**	**0.89**	**0.00**
CD28	Ramos 2016	4 / 7	0.57	0.23	0.86	0.71
CD28	Brudno 2018	15 / 26	0.58	0.39	0.75	0.43
CD28	Li 2018	26 / 28	0.93	0.76	0.98	0.00
**CD28**		**45 / 61**	**0.72**	**0.45**	**0.90**	**0.11**
**Overall**		**254 / 317**	**0.80**	**0.70**	**0.87**	**0.00**

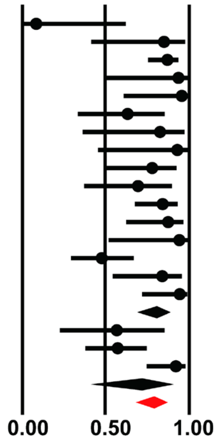

F. CARs

2nd generation	Guo 2016	0 / 5	0.08	0.01	0.62	0.10
2nd generation	Ramos 2016	4 / 7	0.57	0.23	0.86	0.71
2nd generation	Brudno 2018	15 / 26	0.58	0.39	0.75	0.43
2nd generation	Shah 2018	6 / 7	0.86	0.42	0.98	0.10
2nd generation	Zhao 2018	50 / 57	0.88	0.76	0.94	0.00
2nd generation	Mailankody 2018	8 / 8	0.94	0.50	1.00	0.05
2nd generation	Jiang 2018	13 / 13	0.96	0.62	1.00	0.02
2nd generation	Mailankody 2018	7 / 11	0.64	0.34	0.86	0.37
2nd generation	Gregory 2018	5 / 6	0.83	0.37	0.98	0.14
2nd generation	Green 2018	7 / 7	0.94	0.46	1.00	0.06
2nd generation	Liu 2018	11 / 14	0.79	0.51	0.93	0.05
2nd generation	Garfall 2018	7 / 10	0.70	0.38	0.90	0.22
2nd generation	Baumeister 2018	0 / 5	0.08	0.01	0.62	0.10
2nd generation	Li 2018	26 / 28	0.93	0.76	0.98	0.00
2nd generation	Raje 2019	28 / 33	0.85	0.68	0.94	0.00
2nd generation	Xu 2019	15 / 17	0.88	0.63	0.97	0.01
2nd generation	Li 2019	9 / 9	0.95	0.53	1.00	0.04
2nd generation	Cohen 2019	12 / 25	0.48	0.30	0.67	0.84
2nd generation	Han 2019	11 / 13	0.85	0.55	0.96	0.03
2nd generation	Yan 2019	20 / 21	0.95	0.73	0.99	0.00
**2nd generation**		**254 / 322**	**0.78**	**0.68**	**0.86**	**0.00**
3rd generation	Yan 2017	4 / 5	0.80	0.31	0.97	0.21
3rd generation	Popat 2019	6 / 11	0.55	0.27	0.80	0.76
**3rd generation**		**10 / 16**	**0.65**	**0.26**	**0.91**	**0.47**
**Overall**		**264 / 338**	**0.77**	**0.67**	**0.85**	**0.00**

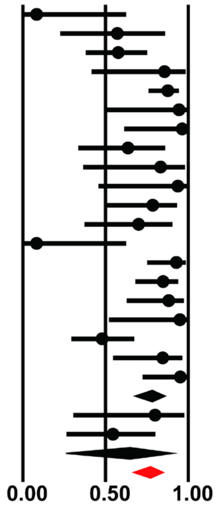

G. Antigen target

BCMA	Brudno 2018	15 / 26	0.58	0.39	0.75	0.43
BCMA	Shah 2018	6 / 7	0.86	0.42	0.98	0.10
BCMA	Zhao 2018	50 / 57	0.88	0.76	0.94	0.00
BCMA	Mailankody 2018	8 / 8	0.94	0.50	1.00	0.05
BCMA	Jiang 2018	13 / 13	0.96	0.62	1.00	0.02
BCMA	Mailankody 2018	7 / 11	0.64	0.34	0.86	0.37
BCMA	Gregory 2018	5 / 6	0.83	0.37	0.98	0.14
BCMA	Green 2018	7 / 7	0.94	0.46	1.00	0.06
BCMA	Liu 2018	11 / 14	0.79	0.51	0.93	0.05
BCMA	Li 2018	26 / 28	0.93	0.76	0.98	0.00
BCMA	Raje 2019	28 / 33	0.85	0.68	0.94	0.00
BCMA	Xu 2019	15 / 17	0.88	0.63	0.97	0.01
BCMA	Li 2019	9 / 9	0.95	0.53	1.00	0.04
BCMA	Cohen 2019	12 / 25	0.48	0.30	0.67	0.84
BCMA	Han 2019	11 / 13	0.85	0.55	0.96	0.03
**BCMA**		**223 / 274**	**0.82**	**0.72**	**0.89**	**0.00**
Non-BCMA	Guo 2016	0 / 5	0.08	0.01	0.62	0.10
Non-BCMA	Ramos 2016	4 / 7	0.57	0.23	0.86	0.71
Non-BCMA	Garfall 2018	7 / 10	0.70	0.38	0.90	0.22
Non-BCMA	Baumeister 2018	0 / 5	0.08	0.01	0.62	0.10
**Non-BCMA**		**11 / 27**	**0.43**	**0.18**	**0.72**	**0.65**
**Overall**		**234 / 301**	**0.67**	**0.26**	**0.92**	**0.43**

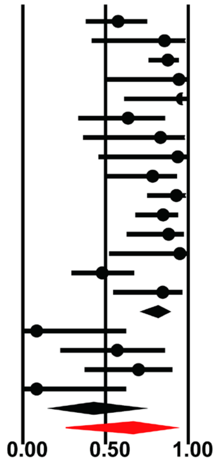

H. CAR-T regimen

Anti-BCMA contained	Yan 2017	4 / 5	0.80	0.31	0.97	0.21
Anti-BCMA contained	Brudno 2018	15 / 26	0.58	0.39	0.75	0.43
Anti-BCMA contained	Shah 2018	6 / 7	0.86	0.42	0.98	0.10
Anti-BCMA contained	Zhao 2018	50 / 57	0.88	0.76	0.94	0.00
Anti-BCMA contained	Mailankody 2018	8 / 8	0.94	0.50	1.00	0.05
Anti-BCMA contained	Jiang 2018	13 / 13	0.96	0.62	1.00	0.02
Anti-BCMA contained	Mailankody 2018	7 / 11	0.64	0.34	0.86	0.37
Anti-BCMA contained	Gregory 2018	5 / 6	0.83	0.37	0.98	0.14
Anti-BCMA contained	Green 2018	7 / 7	0.94	0.46	1.00	0.06
Anti-BCMA contained	Liu 2018	11 / 14	0.79	0.51	0.93	0.05
Anti-BCMA contained	Li 2018	26 / 28	0.93	0.76	0.98	0.00
Anti-BCMA contained	Raje 2019	28 / 33	0.85	0.68	0.94	0.00
Anti-BCMA contained	Xu 2019	15 / 17	0.88	0.63	0.97	0.01
Anti-BCMA contained	Li 2019	9 / 9	0.95	0.53	1.00	0.04
Anti-BCMA contained	Cohen 2019	12 / 25	0.48	0.30	0.67	0.84
Anti-BCMA contained	Han 2019	11 / 13	0.85	0.55	0.96	0.03
Anti-BCMA contained	Yan 2019	20 / 21	0.95	0.73	0.99	0.00
Anti-BCMA contained	Li 2019	10 / 12	0.83	0.52	0.96	0.04
Anti-BCMA contained	Popat 2019	6 / 11	0.55	0.27	0.80	0.76
**Anti-BCMA contained**		**263 / 323**	**0.81**	**0.73**	**0.88**	**0.00**
Anti-BCMA uncontained	Guo 2016	0 / 5	0.08	0.01	0.62	0.10
Anti-BCMA uncontained	Ramos 2016	4 / 7	0.57	0.23	0.86	0.71
Anti-BCMA uncontained	Garfall 2018	7 / 10	0.70	0.38	0.90	0.22
Anti-BCMA uncontained	Baumeister 2018	0 / 5	**0.08**	0.01	0.62	0.10
**Anti-BCMA uncontained**		**11 / 27**	**0.43**	**0.18**	**0.72**	**0.66**
**Overall**		**274 / 350**	**0.67**	**0.27**	**0.92**	**0.42**

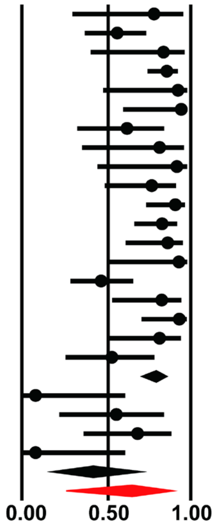

I. CAR-T therapy mode

Dual target	Yan 2017	4 / 5	0.80	0.31	0.97	0.21
Dual target	Yan 2019	20 / 21	0.95	0.73	0.99	0.00
Dual target	Li 2019	10 / 12	0.83	0.52	0.96	0.04
Dual target	Popat 2019	6 / 11	0.55	0.27	0.80	0.76
**Dual target**		**40 / 49**	**0.80**	**0.55**	**0.93**	**0.02**
Single target	Guo 2016	0 / 5	0.08	0.01	0.62	0.10
Single target	Ramos 2016	4 / 7	0.57	0.23	0.86	0.71
Single target	Brudno 2018	15 / 26	0.58	0.39	0.75	0.43
Single target	Shah 2018	6 / 7	0.86	0.42	0.98	0.10
Single target	Zhao 2018	50 / 57	0.88	0.76	0.94	0.00
Single target	Mailankody 2018	8 / 8	0.94	0.50	1.00	0.05
Single target	Jiang 2018	13 / 13	0.96	0.62	1.00	0.02
Single target	Mailankody 2018	7 / 11	0.64	0.34	0.86	0.37
Single target	Gregory 2018	5 / 6	0.83	0.37	0.98	0.14
Single target	Green 2018	7 / 7	0.94	0.46	1.00	0.06
Single target	Liu 2018	11 / 14	0.79	0.51	0.93	0.05
Single target	Li 2018	26 / 28	0.93	0.76	0.98	0.00
Single target	Garfall 2018	7 / 10	0.70	0.38	0.90	0.22
Single target	Baumeister 2018	0 / 5	0.08	0.01	0.62	0.10
Single target	Raje 2019	28 / 33	0.85	0.68	0.94	0.00
Single target	Xu 2019	15 / 17	0.88	0.63	0.97	0.01
Single target	Li 2019	9 / 9	0.95	0.53	1.00	0.04
Single target	Cohen 2019	12 / 25	0.48	0.30	0.67	0.84
Single target	Han 2019	11 / 13	0.85	0.55	0.96	0.03
**Single target**		**234 / 301**	**0.77**	**0.66**	**0.85**	**0.00**
**Overall**		**274 / 350**	**0.77**	**0.68**	**0.85**	**0.00**

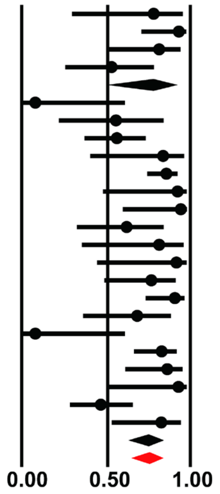

Figure S2 Subgroup analysis of CRS grade 3-4 for RRMM with CAR-T.

A.

Group by: Country	Study name	Total	Event rate	Lower limit	Upper limit	p-Value
Eastern	Guo 2016	0 / 5	0.08	0.01	0.62	0.10
Eastern	Yan 2017	1 / 8	0.13	0.02	0.54	0.07
Eastern	Zhao 2018	4 / 57	0.07	0.03	0.17	0.00
Eastern	Liu 2018	0 / 14	0.03	0.00	0.37	0.02
Eastern	Li 2018	4 / 28	0.14	0.05	0.32	0.00
Eastern	Xu 2019	7 / 17	0.41	0.21	0.65	0.47
Eastern	Li 2019	0 / 9	0.05	0.00	0.47	0.04
Eastern	Han 2019	2 / 16	0.13	0.03	0.39	0.01
Eastern	Yan 2019	1 / 21	0.05	0.01	0.27	0.00
Eastern	Li 2019	4 / 12	0.33	0.13	0.62	0.26
**Eastern**		**23 / 187**	**0.14**	**0.08**	**0.24**	**0.00**
Western	Ramos 2016	0 / 7	0.06	0.00	0.54	0.06
Western	Brudno 2018	6 / 16	0.38	0.18	0.62	0.32
Western	Shah 2018	1 / 8	0.13	0.02	0.54	0.07
Western	Mailankody 2018	0 / 8	0.06	0.00	0.50	0.05
Western	Jiang 2018	1 / 13	0.08	0.01	0.39	0.02
Western	Mailankody 2018	2 / 11	0.18	0.05	0.51	0.05
Western	Gregory 2018	0 / 6	0.07	0.00	0.58	0.08
Western	Green 2018	0 / 7	0.06	0.00	0.54	0.06
Western	Garfall 2018	0 / 10	0.05	0.00	0.45	0.04
Western	Baumeister 2018	0 / 5	0.08	0.01	0.62	0.10
Western	Raje 2019	2 / 33	0.06	0.02	0.21	0.00
Western	Cohen 2019	8 / 25	0.32	0.17	0.52	0.08
Western	Popat 2019	0 / 11	0.04	0.00	0.42	0.03
**Western**		**20 / 160**	**0.14**	**0.08**	**0.23**	**0.00**
**Overall**		**43 / 347**	**0.14**	**0.09**	**0.21**	**0.00**

Statistics for each study

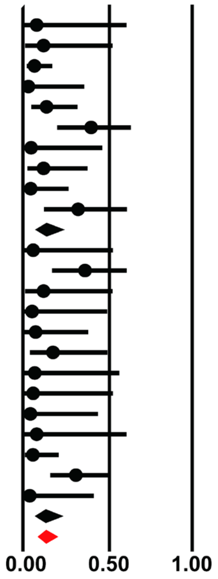

B.

Trial site	Study name	Total	Event rate	Lower limit	Upper limit	p-Value
Multicenter	Ramos 2016	0 / 7	0.06	0.00	0.54	0.06
Multicenter	Brudno 2018	6 / 16	0.38	0.18	0.62	0.32
Multicenter	Shah 2018	1 / 8	0.13	0.02	0.54	0.07
Multicenter	Zhao 2018	4 / 57	0.07	0.03	0.17	0.00
Multicenter	Mailankody 2018	0 / 8	0.06	0.00	0.50	0.05
Multicenter	Jiang 2018	1 / 13	0.08	0.01	0.39	0.02
Multicenter	Gregory 2018	0 / 6	0.07	0.00	0.58	0.08
Multicenter	Liu 2018	0 / 14	0.03	0.00	0.37	0.02
Multicenter	Raje 2019	2 / 33	0.06	0.02	0.21	0.00
Multicenter	Xu 2019	7 / 17	0.41	0.21	0.65	0.47
Multicenter	Popat 2019	0 / 11	0.04	0.00	0.42	0.03
**Multicenter**		**21 / 190**	**0.13**	**0.07**	**0.23**	**0.00**
Single center	Guo 2016	0 / 5	0.08	0.01	0.62	0.10
Single center	Yan 2017	1 / 8	0.13	0.02	0.54	0.07
Single center	Mailankody 2018	2 / 11	0.18	0.05	0.51	0.05
Single center	Green 2018	0 / 7	0.06	0.00	0.54	0.06
Single center	Li 2018	4 / 28	0.14	0.05	0.32	0.00
Single center	Garfall 2018	0 / 10	0.05	0.00	0.45	0.04
Single center	Baumeister 2018	0 / 5	0.08	0.01	0.62	0.10
Single center	Li 2019	0 / 9	0.05	0.00	0.47	0.04
Single center	Cohen 2019	8 / 25	0.32	0.17	0.52	0.08
Single center	Han 2019	2 / 16	0.13	0.03	0.39	0.01
Single center	Yan 2019	1 / 21	0.05	0.01	0.27	0.00
Single center	Li 2019	4 / 12	0.33	0.13	0.62	0.26
**Single center**		**22 / 157**	**0.15**	**0.09**	**0.25**	**0.00**
**Overall**		**43 / 347**	**0.14**	**0.09**	**0.21**	**0.00**

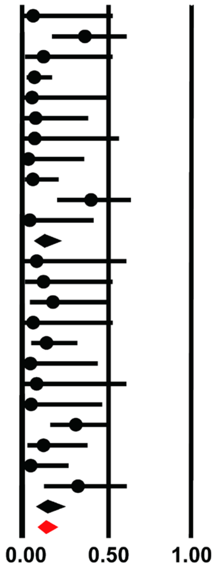

C.

Trial status	Study name	Total	Event rate	Lower limit	Upper limit	p-Value
Completed	Brudno 2018	6 / 16	0.38	0.18	0.62	0.32
Completed	Zhao 2018	4 / 57	0.07	0.03	0.17	0.00
Completed	Xu 2019	7 / 17	0.41	0.21	0.65	0.47
Completed	Garfall 2018	0 / 10	0.05	0.00	0.45	0.04
Completed	Guo 2016	0 / 5	0.08	0.01	0.62	0.10
Completed	Baumeister 2018	0 / 5	0.08	0.01	0.62	0.10
Completed	Yan 2019	1 / 21	0.05	0.01	0.27	0.00
**Completed**		**18 / 131**	**0.17**	**0.09**	**0.30**	**0.00**
Ongoing	Shah 2018	1 / 8	0.13	0.02	0.54	0.07
Ongoing	Mailankody 2018	0 / 8	0.06	0.00	0.50	0.05
Ongoing	Jiang 2018	1 / 13	0.08	0.01	0.39	0.02
Ongoing	Mailankody 2018	2 / 11	0.18	0.05	0.51	0.05
Ongoing	Gregory 2018	0 / 6	0.07	0.00	0.58	0.08
Ongoing	Green 2018	0 / 7	0.06	0.00	0.54	0.06
Ongoing	Liu 2018	0 / 14	0.03	0.00	0.37	0.02
Ongoing	Li 2018	4 / 28	0.14	0.05	0.32	0.00
Ongoing	Raje 2019	2 / 33	0.06	0.02	0.21	0.00
Ongoing	Li 2019	0 / 9	0.05	0.00	0.47	0.04
Ongoing	Cohen 2019	8 / 25	0.32	0.17	0.52	0.08
Ongoing	Han 2019	2 / 16	0.13	0.03	0.39	0.01
Ongoing	Ramos 2016	0 / 7	0.06	0.00	0.54	0.06
Ongoing	Yan 2017	1 / 8	0.13	0.02	0.54	0.07
Ongoing	Li 2019	4 / 12	0.33	0.13	0.62	0.26
Ongoing	Popat 2019	0 / 11	0.04	0.00	0.42	0.03
**Ongoing**		**25 / 216**	**0.13**	**0.08**	**0.21**	**0.00**
**Overall**		**43 / 347**	**0.14**	**0.10**	**0.21**	**0.00**

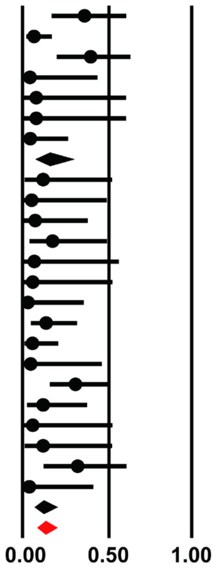

D. scFv origin

Human	Guo 2016	0 / 5	0.08	0.01	0.62	0.10
Human	Mailankody 2018	0 / 8	0.06	0.00	0.50	0.05
Human	Jiang 2018	1 / 13	0.08	0.01	0.39	0.02
Human	Mailankody 2018	2 / 11	0.18	0.05	0.51	0.05
Human	Gregory 2018	0 / 6	0.07	0.00	0.58	0.08
Human	Green 2018	0 / 7	0.06	0.00	0.54	0.06
Human	Li 2019	0 / 9	0.05	0.00	0.47	0.04
Human	Cohen 2019	8 / 25	0.32	0.17	0.52	0.08
**Human**		**11 / 84**	**0.14**	**0.06**	**0.27**	**0.00**
Non-human	Ramos 2016	0 / 7	0.06	0.00	0.54	0.06
Non-human	Yan 2017	1 / 8	0.13	0.02	0.54	0.07
Non-human	Brudno 2018	6 / 16	0.38	0.18	0.62	0.32
Non-human	Shah 2018	1 / 8	0.13	0.02	0.54	0.07
Non-human	Zhao 2018	4 / 57	0.07	0.03	0.17	0.00
Non-human	Liu 2018	0 / 14	0.03	0.00	0.37	0.02
Non-human	Li 2018	4 / 28	0.14	0.05	0.32	0.00
Non-human	Raje 2019	2 / 33	0.06	0.02	0.21	0.00
Non-human	Xu 2019	7 / 17	0.41	0.21	0.65	0.47
Non-human	Han 2019	2 / 16	0.13	0.03	0.39	0.01
Non-human	Yan 2019	1 / 21	0.05	0.01	0.27	0.00
**Non-human**		**28 / 225**	**0.14**	**0.08**	**0.23**	**0.00**
**Overall**		**39 / 309**	**0.14**	**0.09**	**0.21**	**0.00**

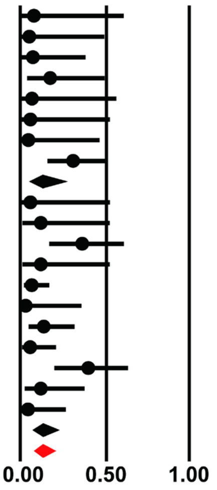

E. co-stimulatory domain

4-1BB	Guo 2016	0 / 5	0.08	0.01	0.62	0.10
4-1BB	Shah 2018	1 / 8	0.13	0.02	0.54	0.07
4-1BB	Zhao 2018	4 / 57	0.07	0.03	0.17	0.00
4-1BB	Mailankody 2018	0 / 8	0.06	0.00	0.50	0.05
4-1BB	Jiang 2018	1 / 13	0.08	0.01	0.39	0.02
4-1BB	Mailankody 2018	2 / 11	0.18	0.05	0.51	0.05
4-1BB	Gregory 2018	0 / 6	0.07	0.00	0.58	0.08
4-1BB	Green 2018	0 / 7	0.06	0.00	0.54	0.06
4-1BB	Liu 2018	0 / 14	0.03	0.00	0.37	0.02
4-1BB	Garfall 2018	0 / 10	0.05	0.00	0.45	0.04
4-1BB	Raje 2019	2 / 33	0.06	0.02	0.21	0.00
4-1BB	Xu 2019	7 / 17	0.41	0.21	0.65	0.47
4-1BB	Li 2019	0 / 9	0.05	0.00	0.47	0.04
4-1BB	Cohen 2019	8 / 25	0.32	0.17	0.52	0.08
4-1BB	Han 2019	2 / 16	0.13	0.03	0.39	0.01
4-1BB	Yan 2019	1 / 21	0.05	0.01	0.27	0.00
**4-1BB**		**28 / 260**	**0.12**	**0.07**	**0.19**	**0.00**
CD28	Ramos 2016	0 / 7	0.06	0.00	0.54	0.06
CD28	Brudno 2018	6 / 16	0.38	0.18	0.62	0.32
CD28	Li 2018	4 / 28	0.14	0.05	0.32	0.00
**CD28**		**10 / 51**	**0.21**	**0.08**	**0.44**	**0.02**
**Overall**		**38 / 311**	**0.14**	**0.08**	**0.22**	**0.00**

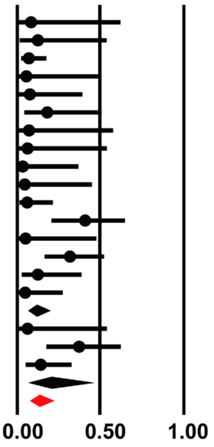

F. CARs

2nd generation	Guo 2016	0 / 5	0.08	0.01	0.62	0.10
2nd generation	Ramos 2016	0 / 7	0.06	0.00	0.54	0.06
2nd generation	Brudno 2018	6 / 16	0.38	0.18	0.62	0.32
2nd generation	Shah 2018	1 / 8	0.13	0.02	0.54	0.07
2nd generation	Zhao 2018	4 / 57	0.07	0.03	0.17	0.00
2nd generation	Mailankody 2018	0 / 8	0.06	0.00	0.50	0.05
2nd generation	Jiang 2018	1 / 13	0.08	0.01	0.39	0.02
2nd generation	Mailankody 2018	2 / 11	0.18	0.05	0.51	0.05
2nd generation	Gregory 2018	0 / 6	0.07	0.00	0.58	0.08
2nd generation	Green 2018	0 / 7	0.06	0.00	0.54	0.06
2nd generation	Liu 2018	0 / 14	0.03	0.00	0.37	0.02
2nd generation	Li 2018	4 / 28	0.14	0.05	0.32	0.00
2nd generation	Garfall 2018	0 / 10	0.05	0.00	0.45	0.04
2nd generation	Baumeister 2018	0 / 5	0.08	0.01	0.62	0.10
2nd generation	Raje 2019	2 / 33	0.06	0.02	0.21	0.00
2nd generation	Xu 2019	7 / 17	0.41	0.21	0.65	0.47
2nd generation	Li 2019	0 / 9	0.05	0.00	0.47	0.04
2nd generation	Cohen 2019	8 / 25	0.32	0.17	0.52	0.08
2nd generation	Han 2019	2 / 16	0.13	0.03	0.39	0.01
2nd generation	Yan 2019	1 / 21	0.05	0.01	0.27	0.00
**2nd generation**		**38 / 316**	**0.14**	**0.09**	**0.20**	**0.00**
3rd generation	Yan 2017	1 / 8	0.13	0.02	0.54	0.07
3rd generation	Popat 2019	0 / 11	0.04	0.00	0.42	0.03
**3rd generation**		**1 / 19**	**0.08**	**0.01**	**0.37**	**0.01**
**Overall**		**39 / 335**	**0.13**	**0.09**	**0.20**	**0.00**

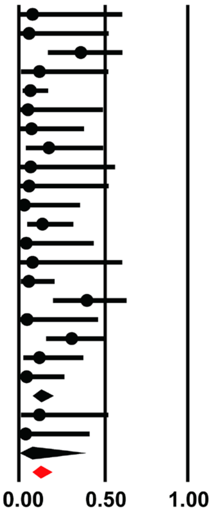

G. Antigen target

BCMA	Brudno 2018	6 / 16	0.38	0.18	0.62	0.32
BCMA	Shah 2018	1 / 8	0.13	0.02	0.54	0.07
BCMA	Zhao 2018	4 / 57	0.07	0.03	0.17	0.00
BCMA	Mailankody 2018	0 / 8	0.06	0.00	0.50	0.05
BCMA	Jiang 2018	1 / 13	0.08	0.01	0.39	0.02
BCMA	Mailankody 2018	2 / 11	0.18	0.05	0.51	0.05
BCMA	Gregory 2018	0 / 6	0.07	0.00	0.58	0.08
BCMA	Green 2018	0 / 7	0.06	0.00	0.54	0.06
BCMA	Liu 2018	0 / 14	0.03	0.00	0.37	0.02
BCMA	Li 2018	4 / 28	0.14	0.05	0.32	0.00
BCMA	Raje 2019	2 / 33	0.06	0.02	0.21	0.00
BCMA	Xu 2019	7 / 17	0.41	0.21	0.65	0.47
BCMA	Li 2019	0 / 9	0.05	0.00	0.47	0.04
BCMA	Cohen 2019	8 / 25	0.32	0.17	0.52	0.08
BCMA	Han 2019	2 / 16	0.13	0.03	0.39	0.01
**BCMA**		**37 / 268**	**0.16**	**0.10**	**0.23**	**0.00**
Non-BCMA	Guo 2016	0 / 5	0.08	0.01	0.62	0.10
Non-BCMA	Ramos 2016	0 / 7	0.06	0.00	0.54	0.06
Non-BCMA	Garfall 2018	0 / 10	0.05	0.00	0.45	0.04
Non-BCMA	Baumeister 2018	0 / 5	0.08	0.01	0.62	0.10
**Non-BCMA**		**0 / 27**	**0.07**	**0.02**	**0.25**	**0.00**
**Overall**		**37 / 295**	**0.13**	**0.07**	**0.24**	**0.00**

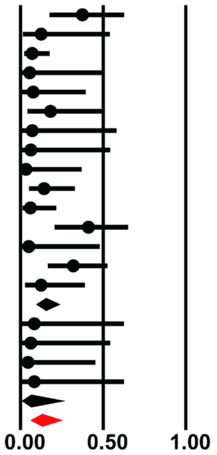

H. CAR-T regimen

Anti-BCMA contained	Yan 2017	1 / 8	0.13	0.02	0.54	0.07
Anti-BCMA contained	Brudno 2018	6 / 16	0.38	0.18	0.62	0.32
Anti-BCMA contained	Shah 2018	1 / 8	0.13	0.02	0.54	0.07
Anti-BCMA contained	Zhao 2018	4 / 57	0.07	0.03	0.17	0.00
Anti-BCMA contained	Mailankody 2018	0 / 8	0.06	0.00	0.50	0.05
Anti-BCMA contained	Jiang 2018	1 / 13	0.08	0.01	0.39	0.02
Anti-BCMA contained	Mailankody 2018	2 / 11	0.18	0.05	0.51	0.05
Anti-BCMA contained	Gregory 2018	0 / 6	0.07	0.00	0.58	0.08
Anti-BCMA contained	Green 2018	0 / 7	0.06	0.00	0.54	0.06
Anti-BCMA contained	Liu 2018	0 / 14	0.03	0.00	0.37	0.02
Anti-BCMA contained	Li 2018	4 / 28	0.14	0.05	0.32	0.00
Anti-BCMA contained	Raje 2019	2 / 33	0.06	0.02	0.21	0.00
Anti-BCMA contained	Xu 2019	7 / 17	0.41	0.21	0.65	0.47
Anti-BCMA contained	Li 2019	0 / 9	0.05	0.00	0.47	0.04
Anti-BCMA contained	Cohen 2019	8 / 25	0.32	0.17	0.52	0.08
Anti-BCMA contained	Han 2019	2 / 16	0.13	0.03	0.39	0.01
Anti-BCMA contained	Yan 2019	1 / 21	0.05	0.01	0.27	0.00
Anti-BCMA contained	Li 2019	4 / 12	0.33	0.13	0.62	0.26
Anti-BCMA contained	Popat 2019	0 / 11	0.04	0.00	0.42	0.03
**Anti-BCMA contained**		**43 / 320**	**0.15**	**0.10**	**0.22**	**0.00**
Anti-BCMA uncontained	Guo 2016	0 / 5	0.08	0.01	0.62	0.10
Anti-BCMA uncontained	Ramos 2016	0 / 7	0.06	0.00	0.54	0.06
Anti-BCMA uncontained	Garfall 2018	0 / 10	0.05	0.00	0.45	0.04
Anti-BCMA uncontained	Baumeister 2018	0 / 5	0.08	0.01	0.62	0.10
**Anti-BCMA uncontained**		**0 / 27**	**0.07**	**0.02**	**0.25**	**0.00**
**Overall**		**43 / 347**	**0.13**	**0.07**	**0.24**	**0.00**

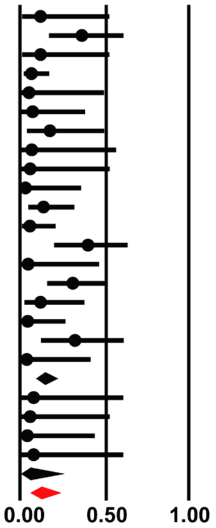

I. CAR-T therapy mode

Dual target	Yan 2017	1 / 8	0.13	0.02	0.54	0.07
Dual target	Yan 2019	1 / 21	0.05	0.01	0.27	0.00
Dual target	Li 2019	4 / 12	0.33	0.13	0.62	0.26
Dual target	Popat 2019	0 / 11	0.04	0.00	0.42	0.03
**Dual target**		**6 / 52**	**0.14**	**0.05**	**0.34**	**0.00**
Single target	Guo 2016	0 / 5	0.08	0.01	0.62	0.10
Single target	Ramos 2016	0 / 7	0.06	0.00	0.54	0.06
Single target	Brudno 2018	6 / 16	0.38	0.18	0.62	0.32
Single target	Shah 2018	1 / 8	0.13	0.02	0.54	0.07
Single target	Zhao 2018	4 / 57	0.07	0.03	0.17	0.00
Single target	Mailankody 2018	0 / 8	0.06	0.00	0.50	0.05
Single target	Jiang 2018	1 / 13	0.08	0.01	0.39	0.02
Single target	Mailankody 2018	2 / 11	0.18	0.05	0.51	0.05
Single target	Gregory 2018	0 / 6	0.07	0.00	0.58	0.08
Single target	Green 2018	0 / 7	0.06	0.00	0.54	0.06
Single target	Liu 2018	0 / 14	0.03	0.00	0.37	0.02
Single target	Li 2018	4 / 28	0.14	0.05	0.32	0.00
Single target	Garfall 2018	0 / 10	0.05	0.00	0.45	0.04
Single target	Baumeister 2018	0 / 5	0.08	0.01	0.62	0.10
Single target	Raje 2019	2 / 33	0.06	0.02	0.21	0.00
Single target	Xu 2019	7 / 17	0.41	0.21	0.65	0.47
Single target	Li 2019	0 / 9	0.05	0.00	0.47	0.04
Single target	Cohen 2019	8 / 25	0.32	0.17	0.52	0.08
Single target	Han 2019	2 / 16	0.13	0.03	0.39	0.01
**Single target**		**37 / 295**	**0.14**	**0.09**	**0.21**	**0.00**
**Overall**		**43 / 347**	**0.14**	**0.09**	**0.21**	**0.00**

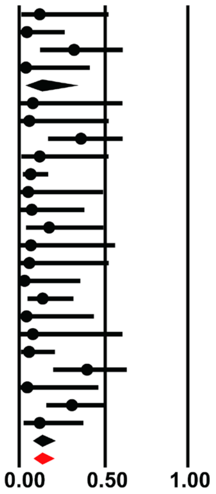

Figure S3 Subgroup analysis of NT for RRMM with CAR-T.

A.

Group by	Study name		Statistics for each study				Event rate and 95% CI
Country		Total	Event rate	Lower limit	Upper limit	p-Value	
Eastern	Guo 2016	0 / 5	0.08	0.01	0.62	0.10	
Eastern	Yan 2017	0 / 8	0.06	0.00	0.50	0.05	
Eastern	Zhao 2018	1 / 57	0.02	0.00	0.11	0.00	
Eastern	Liu 2018	1 / 14	0.07	0.01	0.37	0.01	
Eastern	Li 2019	0 / 12	0.04	0.00	0.40	0.03	
Eastern	Yan 2019	2 / 21	0.10	0.02	0.31	0.00	
**Eastern**		**4 / 117**	**0.06**	**0.02**	**0.13**	**0.00**	
Western	Ramos 2016	0 / 7	0.06	0.00	0.54	0.06	
Western	Brudno 2018	6 / 16	0.38	0.18	0.62	0.32	
Western	Shah 2018	1 / 8	0.13	0.02	0.54	0.07	
Western	Mailankody 2018	3 / 8	0.38	0.13	0.72	0.48	
Western	Jiang 2018	0 / 13	0.04	0.00	0.38	0.02	
Western	Mailankody 2018	1 / 11	0.09	0.01	0.44	0.03	
Western	Gregory 2018	0 / 6	0.07	0.00	0.58	0.08	
Western	Green 2018	0 / 7	0.06	0.00	0.54	0.06	
Western	Garfall 2018	0 / 10	0.05	0.00	0.45	0.04	
Western	Baumeister 2018	0 / 5	0.08	0.01	0.62	0.10	
Western	Raje 2019	14 / 33	0.42	0.27	0.60	0.39	
Western	Cohen 2019	8 / 25	0.32	0.17	0.52	0.08	
Western	Rakesh 2019	0 / 11	0.04	0.00	0.42	0.03	
**Western**		**33 / 160**	**0.23**	**0.15**	**0.34**	**0.00**	
**Overall**		**37 / 277**	**0.12**	**0.03**	**0.41**	**0.02**	

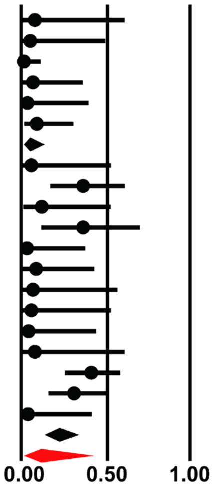

B.

Trial site	Study name	Total	Event rate	Lower limit	Upper limit	p-Value
Multicenter	Ramos 2016	0 / 7	0.06	0.00	0.54	0.06
Multicenter	Brudno 2018	6 / 16	0.38	0.18	0.62	0.32
Multicenter	Shah 2018	1 / 8	0.13	0.02	0.54	0.07
Multicenter	Zhao 2018	1 / 57	0.02	0.00	0.11	0.00
Multicenter	Mailankody 2018	3 / 8	0.38	0.13	0.72	0.48
Multicenter	Jiang 2018	0 / 13	0.04	0.00	0.38	0.02
Multicenter	Gregory 2018	0 / 6	0.07	0.00	0.58	0.08
Multicenter	Liu 2018	1 / 14	0.07	0.01	0.37	0.01
Multicenter	Raje 2019	14 / 33	0.42	0.27	0.60	0.39
Multicenter	Popat 2019	0 / 11	0.04	0.00	0.42	0.03
**Multicenter**		**26 / 173**	**0.15**	**0.07**	**0.29**	**0.00**
Single center	Guo 2016	0 / 5	0.08	0.01	0.62	0.10
Single center	Yan 2017	0 / 8	0.06	0.00	0.50	0.05
Single center	Mailankody 2018	1 / 11	0.09	0.01	0.44	0.03
Single center	Green 2018	0 / 7	0.06	0.00	0.54	0.06
Single center	Garfall 2018	0 / 10	0.05	0.00	0.45	0.04
Single center	Baumeister 2018	0 / 5	0.08	0.01	0.62	0.10
Single center	Cohen 2019	8 / 25	0.32	0.17	0.52	0.08
Single center	Yan 2019	2 / 21	0.10	0.02	0.31	0.00
Single center	Li 2019	0 / 12	0.04	0.00	0.40	0.03
**Single center**		**11 / 104**	**0.10**	**0.04**	**0.23**	**0.00**
**Overall**		**37 / 277**	**0.13**	**0.08**	**0.22**	**0.00**

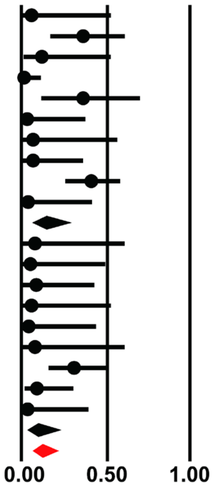

C.

Trial status	Study name	Total	Event rate	Lower limit	Upper limit	p-Value
Completed	Brudno 2018	6 / 16	0.38	0.18	0.62	0.32
Completed	Zhao 2018	1 / 57	0.02	0.00	0.11	0.00
Completed	Garfall 2018	0 / 10	0.05	0.00	0.45	0.04
Completed	Guo 2016	0 / 5	0.08	0.01	0.62	0.10
Completed	Baumeister 2018	0 / 5	0.08	0.01	0.62	0.10
Completed	Yan 2019	2 / 21	0.10	0.02	0.31	0.00
**Completed**		**9 / 114**	**0.10**	**0.04**	**0.25**	**0.00**
Ongoing	Shah 2018	1 / 8	0.13	0.02	0.54	0.07
Ongoing	Mailankody 2018	3 / 8	0.38	0.13	0.72	0.48
Ongoing	Jiang 2018	0 / 13	0.04	0.00	0.38	0.02
Ongoing	Mailankody 2018	1 / 11	0.09	0.01	0.44	0.03
Ongoing	Gregory 2018	0 / 6	0.07	0.00	0.58	0.08
Ongoing	Green 2018	0 / 7	0.06	0.00	0.54	0.06
Ongoing	Liu 2018	1 / 14	0.07	0.01	0.37	0.01
Ongoing	Raje 2019	14 / 33	0.42	0.27	0.60	0.39
Ongoing	Cohen 2019	8 / 25	0.32	0.17	0.52	0.08
Ongoing	Ramos 2016	0 / 7	0.06	0.00	0.54	0.06
Ongoing	Yan 2017	0 / 8	0.06	0.00	0.50	0.05
Ongoing	Li 2019	0 / 12	0.04	0.00	0.40	0.03
Ongoing	Popat 2019	0 / 11	0.04	0.00	0.42	0.03
**Ongoing**		**28 / 163**	**0.15**	**0.08**	**0.27**	**0.00**
**Overall**		**37 / 277**	**0.13**	**0.08**	**0.22**	**0.00**

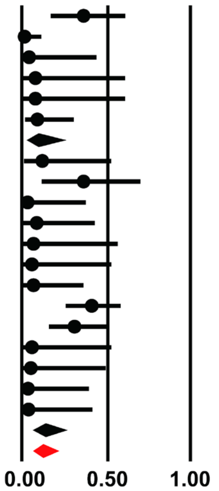

D. **scFv origin**

Human	Guo 2016	0 / 5	0.08	0.01	0.62	0.10
Human	Mailankody 2018	3 / 8	0.38	0.13	0.72	0.48
Human	Jiang 2018	0 / 13	0.04	0.00	0.38	0.02
Human	Mailankody 2018	1 / 11	0.09	0.01	0.44	0.03
Human	Gregory 2018	0 / 6	0.07	0.00	0.58	0.08
Human	Green 2018	0 / 7	0.06	0.00	0.54	0.06
Human	Cohen 2019	8 / 25	0.32	0.17	0.52	0.08
**Human**		**12 / 75**	**0.16**	**0.06**	**0.35**	**0.00**
Non-human	Ramos 2016	0 / 7	0.06	0.00	0.54	0.06
Non-human	Yan 2017	0 / 8	0.06	0.00	0.50	0.05
Non-human	Brudno 2018	6 / 16	0.38	0.18	0.62	0.32
Non-human	Shah 2018	1 / 8	0.13	0.02	0.54	0.07
Non-human	Zhao 2018	1 / 57	0.02	0.00	0.11	0.00
Non-human	Liu 2018	1 / 14	0.07	0.01	0.37	0.01
Non-human	Baumeister 2018	0 / 5	0.08	0.01	0.62	0.10
Non-human	Raje 2019	14 / 33	0.42	0.27	0.60	0.39
Non-human	Yan 2019	2 / 21	0.10	0.02	0.31	0.00
**Non-human**		**25 / 169**	**0.14**	**0.06**	**0.28**	**0.00**
**Overall**		**37 / 244**	**0.15**	**0.08**	**0.25**	**0.00**

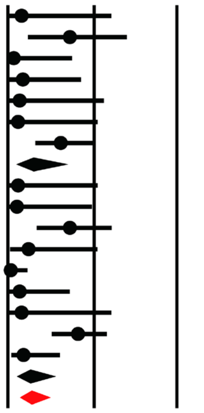

E. **co-stimulatory domain**

4-1BB	Guo 2016	0 / 5	0.08	0.01	0.62	0.10
4-1BB	Shah 2018	1 / 8	0.13	0.02	0.54	0.07
4-1BB	Mailankody 2018	3 / 8	0.38	0.13	0.72	0.48
4-1BB	Jiang 2018	0 / 13	0.04	0.00	0.38	0.02
4-1BB	Mailankody 2018	1 / 11	0.09	0.01	0.44	0.03
4-1BB	Gregory 2018	0 / 6	0.07	0.00	0.58	0.08
4-1BB	Green 2018	0 / 7	0.06	0.00	0.54	0.06
4-1BB	Garfall 2018	0 / 10	0.05	0.00	0.45	0.04
4-1BB	Zhao 2018	1 / 57	0.02	0.00	0.11	0.00
4-1BB	Liu 2018	1 / 14	0.07	0.01	0.37	0.01
4-1BB	Raje 2019	14 / 33	0.42	0.27	0.60	0.39
4-1BB	Cohen 2019	8 / 25	0.32	0.17	0.52	0.08
4-1BB	Yan 2019	2 / 21	0.10	0.02	0.31	0.00
**4-1BB**		**31 / 218**	**0.13**	**0.07**	**0.24**	**0.00**
CD28	Ramos 2016	0 / 7	0.06	0.00	0.54	0.06
CD28	Brudno 2018	6 / 16	0.38	0.18	0.62	0.32
**CD28**		**6 / 23**	**0.25**	**0.05**	**0.66**	**0.22**
**Overall**		**37 / 241**	**0.15**	**0.08**	**0.25**	**0.00**

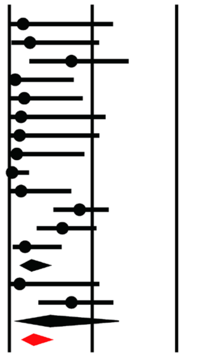

F. **CARs**

2nd generation	Ramos 2016	0 / 7	0.06	0.00	0.54	0.06
2nd generation	Guo 2016	0 / 5	0.08	0.01	0.62	0.10
2nd generation	Brudno 2018	6 / 16	0.38	0.18	0.62	0.32
2nd generation	Shah 2018	1 / 8	0.13	0.02	0.54	0.07
2nd generation	Mailankody 2018	3 / 8	0.38	0.13	0.72	0.48
2nd generation	Jiang 2018	0 / 13	0.04	0.00	0.38	0.02
2nd generation	Mailankody 2018	1 / 11	0.09	0.01	0.44	0.03
2nd generation	Gregory 2018	0 / 6	0.07	0.00	0.58	0.08
2nd generation	Green 2018	0 / 7	0.06	0.00	0.54	0.06
2nd generation	Garfall 2018	0 / 10	0.05	0.00	0.45	0.04
2nd generation	Zhao 2018	1 / 57	0.02	0.00	0.11	0.00
2nd generation	Liu 2018	1 / 14	0.07	0.01	0.37	0.01
2nd generation	Baumeister 2018	0 / 5	0.08	0.01	0.62	0.10
2nd generation	Raje 2019	14 / 33	0.42	0.27	0.60	0.39
2nd generation	Cohen 2019	8 / 25	0.32	0.17	0.52	0.08
2nd generation	Yan 2019	2 / 21	0.10	0.02	0.31	0.00
**2nd generation**		**37 / 246**	**0.15**	**0.09**	**0.25**	**0.00**
3rd generation	Yan 2017	0 / 8	0.06	0.00	0.50	0.05
3rd generation	Rakesh 2019	0 / 11	0.04	0.00	0.42	0.03
**3rd generation**		**0 / 19**	**0.05**	**0.01**	**0.33**	**0.01**
**Overall**		**37 / 265**	**0.14**	**0.07**	**0.25**	**0.00**

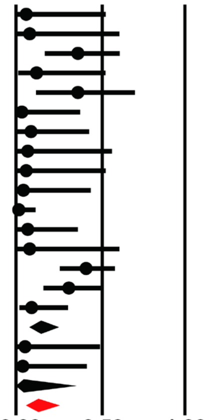

G. Antigen target

BCMA	Brudno 2018	6 / 16	0.38	0.18	0.62	0.32
BCMA	Shah 2018	1 / 8	0.13	0.02	0.54	0.07
BCMA	Zhao 2018	1 / 57	0.02	0.00	0.11	0.00
BCMA	Mailankody 2018	3 / 8	0.38	0.13	0.72	0.48
BCMA	Jiang 2018	0 / 13	0.04	0.00	0.38	0.02
BCMA	Mailankody 2018	1 / 11	0.09	0.01	0.44	0.03
BCMA	Gregory 2018	0 / 6	0.07	0.00	0.58	0.08
BCMA	Green 2018	0 / 7	0.06	0.00	0.54	0.06
BCMA	Liu 2018	1 / 14	0.07	0.01	0.37	0.01
BCMA	Raje 2019	14 / 33	0.42	0.27	0.60	0.39
BCMA	Cohen 2019	8 / 25	0.32	0.17	0.52	0.08
**BCMA**		**35 / 198**	**0.19**	**0.11**	**0.31**	**0.00**
Non-BCMA	Guo 2016	0 / 5	0.08	0.01	0.62	0.10
Non-BCMA	Ramos 2016	0 / 7	0.06	0.00	0.54	0.06
Non-BCMA	Garfall 2018	0 / 10	0.05	0.00	0.45	0.04
Non-BCMA	Baumeister 2018	0 / 5	0.08	0.01	0.62	0.10
**Non-BCMA**		**0 / 27**	**0.07**	**0.01**	**0.26**	**0.00**
**Overall**		**35 / 225**	**0.14**	**0.05**	**0.32**	**0.00**

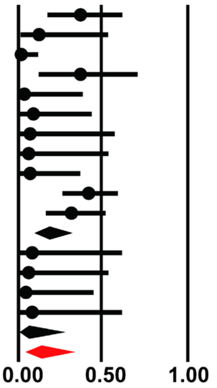

H. CAR-T regimen

Anti-BCMA contained	Yan 2017	0 / 8	0.06	0.00	0.50	0.05
Anti-BCMA contained	Brudno 2018	6 / 16	0.38	0.18	0.62	0.32
Anti-BCMA contained	Shah 2018	1 / 8	0.13	0.02	0.54	0.07
Anti-BCMA contained	Zhao 2018	1 / 57	0.02	0.00	0.11	0.00
Anti-BCMA contained	Mailankody 2018	3 / 8	0.38	0.13	0.72	0.48
Anti-BCMA contained	Jiang 2018	0 / 13	0.04	0.00	0.38	0.02
Anti-BCMA contained	Mailankody 2018	1 / 11	0.09	0.01	0.44	0.03
Anti-BCMA contained	Gregory 2018	0 / 6	0.07	0.00	0.58	0.08
Anti-BCMA contained	Green 2018	0 / 7	0.06	0.00	0.54	0.06
Anti-BCMA contained	Liu 2018	1 / 14	0.07	0.01	0.37	0.01
Anti-BCMA contained	Raje 2019	14 / 33	0.42	0.27	0.60	0.39
Anti-BCMA contained	Cohen 2019	8 / 25	0.32	0.17	0.52	0.08
Anti-BCMA contained	Yan 2019	2 / 21	0.10	0.02	0.31	0.00
Anti-BCMA contained	Li 2019	0 / 12	0.04	0.00	0.40	0.03
Anti-BCMA contained	Popat 2019	0 / 11	0.04	0.00	0.42	0.03
**Anti-BCMA contained**		**37 / 250**	**0.15**	**0.09**	**0.25**	**0.00**
Anti-BCMA uncontained	Guo 2016	0 / 5	0.08	0.01	0.62	0.10
Anti-BCMA uncontained	Ramos 2016	0 / 7	0.06	0.00	0.54	0.06
Anti-BCMA uncontained	Garfall 2018	0 / 10	0.05	0.00	0.45	0.04
Anti-BCMA uncontained	Baumeister 2018	0 / 5	0.08	0.01	0.62	0.10
**Anti-BCMA uncontained**		**0 / 27**	**0.07**	**0.01**	**0.27**	**0.00**
		**37 / 277**	**0.13**	**0.08**	**0.23**	**0.00**

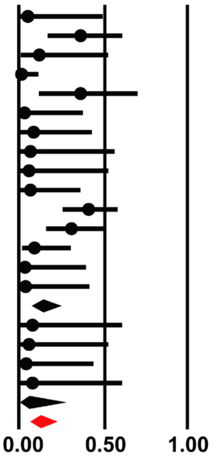

I. CAR-T therapy mode

Dual target	Yan 2017	0 / 8	0.06	0.00	0.50	0.05
Dual target	Yan 2019	2 / 21	0.10	0.02	0.31	0.00
Dual target	Li 2019	0 / 12	0.04	0.00	0.40	0.03
Dual target	Popat 2019	0 / 11	0.04	0.00	0.42	0.03
**Dual target**		**2 / 52**	**0.06**	**0.02**	**0.21**	**0.00**
Single target	Guo 2016	0 / 5	0.08	0.01	0.62	0.10
Single target	Ramos 2016	0 / 7	0.06	0.00	0.54	0.06
Single target	Brudno 2018	6 / 16	0.38	0.18	0.62	0.32
Single target	Shah 2018	1 / 8	0.13	0.02	0.54	0.07
Single target	Zhao 2018	1 / 57	0.02	0.00	0.11	0.00
Single target	Mailankody 2018	3 / 8	0.38	0.13	0.72	0.48
Single target	Jiang 2018	0 / 13	0.04	0.00	0.38	0.02
Single target	Mailankody 2018	1 / 11	0.09	0.01	0.44	0.03
Single target	Gregory 2018	0 / 6	0.07	0.00	0.58	0.08
Single target	Green 2018	0 / 7	0.06	0.00	0.54	0.06
Single target	Liu 2018	1 / 14	0.07	0.01	0.37	0.01
Single target	Garfall 2018	0 / 10	0.05	0.00	0.45	0.04
Single target	Baumeister 2018	0 / 5	0.08	0.01	0.62	0.10
Single target	Raje 2019	14 / 33	0.42	0.27	0.60	0.39
Single target	Cohen 2019	8 / 25	0.32	0.17	0.52	0.08
**Single target**		**35 / 225**	**0.16**	**0.10**	**0.27**	**0.00**
**Overall**		**37 / 277**	**0.12**	**0.05**	**0.27**	**0.00**

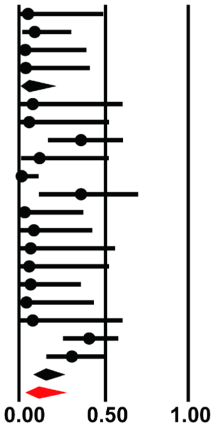

Figure S4:

Funnel plot for RRMM with CAR-T reporting Overall Response (A), Complete Response (B), MRD negativity (C), Relapse at last follow-up (D), Overall Survival at last follow-up (E), CRS (F) and NT (G).

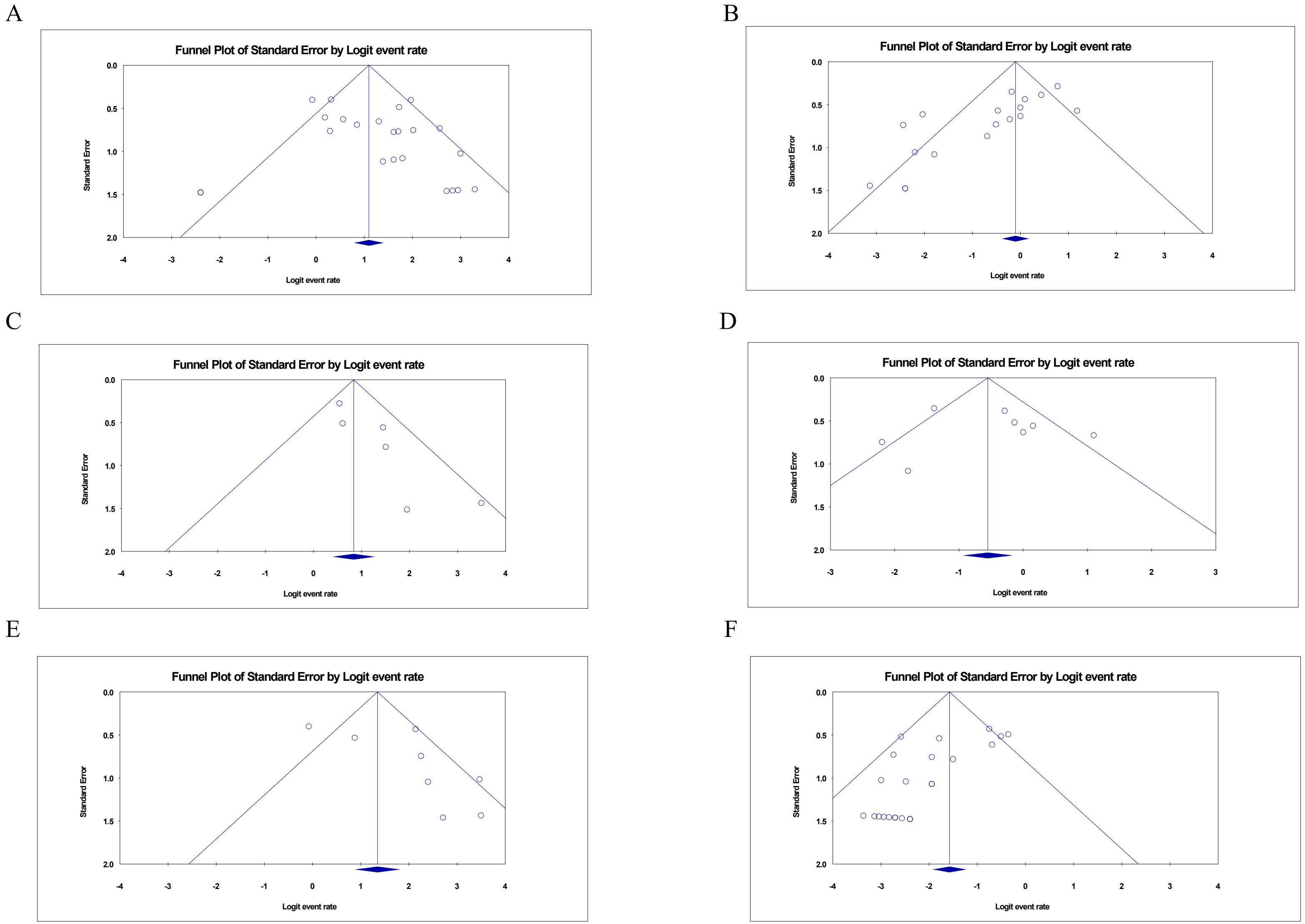

G

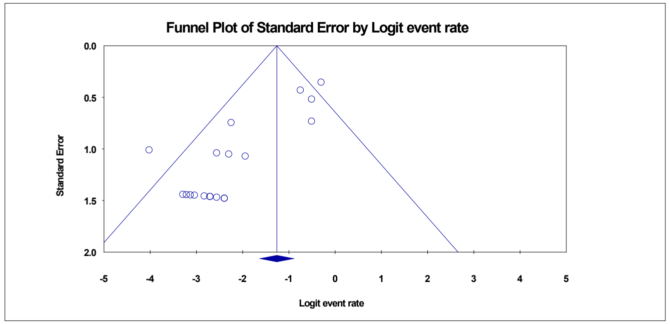

Funnel Plot of Standard Error by Logit event rate
Standard Error
0.0
0.5
1.0
1.5
2.0
-5
-4
-3
-2
-1
0
1
2
3
4
5
Logit event rate
